# Adapt‐on‐demand: Enabling flexible and scalable adaptive radiotherapy through workflow automation

**DOI:** 10.1002/acm2.70612

**Published:** 2026-05-13

**Authors:** Sean Domal, Min Geon Choi, Justin Visak, Ruiqi Li, Taoran Li, Boon‐Keng Kevin Teo, Xiaodong Zhao, Matthew Schmidt, Eric Laugeman, David Parsons, Yang Kyun Park, Shahed Badiyan, David Sher, Mu‐Han Lin

**Affiliations:** ^1^ Department of Radiation Oncology UT Southwestern Medical Center Dallas Texas USA; ^2^ Department of Radiation Oncology University of Pennsylvania Philadelphia Pennsylvania USA; ^3^ Department of Radiation Oncology Washington University in St. Louis St. Louis Missouri USA

**Keywords:** adapt‐on‐demand, adaptive radiotherapy, CT‐guided adaptive radiotherapy, ESAPI, Ethos, Halcyon, workflow automation

## Abstract

**Purpose:**

CT‐guided adaptive radiotherapy (CTgART) offers significant clinical benefits, but its utilization is often limited by resource demands and rigid workflows. This study presents a flexible and scalable “Adapt‐on‐Demand” (AOD) framework that integrates the adaptive capabilities of the Varian Ethos platform with the high‐throughput IGRT delivery of the Varian Halcyon, supported by automated scripting.

**Methods:**

We developed an end‐to‐end dual‐platform workflow that dynamically transitions between ART and IGRT delivery based on patient needs. Key components include Eclipse Scripting API (ESAPI) automation to streamline adapted plan ingestion from the Ethos Treatment Planning and Management System (TPMS) export directory, compatibility conversion for Halcyon delivery, and ARIA chart setup. The framework was evaluated through time‐efficiency analysis and longitudinal tracking of ART utilization and machine throughput. Multi‐institutional deployment was also conducted to validate generalizability.

**Results:**

Automation reduced plan setup time from 45 to 8 min (*p* < 0.001, unpaired *t*‐test), and direct comparison of MLC leaf positions confirmed exact agreement between Ethos‐native and converted plans across all control points. ART utilization on the Ethos platform increased from 17% to 77% following IGRT offloading to Halcyon. The workflow was successfully implemented at two external sites without code modification, demonstrating portability.

**Conclusion:**

This AOD framework enables flexible ART delivery without compromising workflow efficiency. By automating inter‐platform transitions and alleviating adaptive system burden, it improves clinical throughput and broadens patient access to ART. This work also underscores the importance of system interoperability and provides a scalable model adaptable across diverse practice settings.

## 1 INTRODUCTION

Adaptive radiotherapy (ART) represents a major advancement in radiation oncology, allowing for treatment plans to be dynamically modified in response to patient‐specific anatomical changes. By improving target coverage and sparing surrounding organs at risk (OARs), ART has shown the potential to enhance treatment precision and clinical outcomes across multiple disease sites, including prostate, bladder, and abdominal tumors.^1^, ^2^, ^3^, ^4^, ^5^, ^6^, ^7^, ^8^, ^9^

However, the implementation of daily online ART (oART) in routine clinical practice remains challenging. The process requires daily imaging, contour propagation, re‐optimization, and plan quality assurance—all of which contribute to increased on‐table time, staff workload, and resource demands. Emerging clinical evidence suggests that not all patients benefit equally from daily adaptation. For patients with relatively stable anatomy or minimal dosimetric changes, the additional effort associated with daily ART may offer only marginal gains.^10^, ^11^, ^12^, ^13^ These findings underscore the need for flexibility in adaptation frequency, tailoring treatment intensity to patient‐specific needs rather than applying a uniform daily schedule.

The concept of variable‐frequency or “adapt‐on‐demand” treatment has been explored in the literature, including dosimetric studies evaluating the benefit of selective adaptation for specific disease sites.^12^ However, translating this concept into routine clinical practice requires not only dosimetric justification but also robust clinical infrastructure—automated tools, standardized workflows, and inter‐system communication pathways—that can support seamless transitions between adaptive and non‐adaptive delivery. This infrastructure gap represents a significant barrier to broader adoption of flexible ART paradigms.

The Varian Ethos platform (Varian Medical Systems, Palo Alto, CA) provides an integrated CT‐guided adaptive radiotherapy (CTgART) solution featuring high‐quality iterative CBCT imaging (HyperSight), automated contour propagation, intelligent optimization templates, and built‐in plan quality assurance, all designed to streamline adaptive treatment delivery.^5^, ^11^, ^13^ Yet in its current clinical implementation, the Ethos Treatment Planning and Management System (TPMS) requires a full commitment to either an adaptive or IGRT‐based workflow, which limits flexibility. Transitioning between ART and IGRT modes requires revision of the treatment plan within the Ethos TPMS and is logistically burdensome.

In contrast, the Varian Halcyon enables rapid, high‐quality CBCT‐guided imaging and is optimized for efficient, high‐throughput IGRT treatment delivery. However, it lacks native adaptive capabilities.^10^, ^11^, ^12^, ^13^, ^14^, ^15^, ^16^, ^17^, ^18^ The complementary nature of these two platforms presents a unique opportunity: to perform adaptation only when necessary on Ethos while delivering stable fractions on Halcyon using standard IGRT. This hybrid strategy can enhance overall machine throughput, reserve adaptive resources for cases with the greatest clinical benefit, and triage non‐adaptive treatments to Halcyon—thereby maintaining workflow flexibility without consuming valuable adaptive machine time. Importantly, the current automation primarily supports the Ethos‐to‐IGRT direction; transitioning a patient back to Ethos for a new adaptive session requires re‐enrollment in the Ethos TPMS through manual steps.

To realize this vision, it is essential to develop integrated tools and workflows that enable seamless coordination between the two systems, allowing efficient transfer of patients between ART and IGRT. The Eclipse Scripting API (ESAPI) has been proven effective for automating diverse radiotherapy tasks on conventional treatment planning platforms, including knowledge‐based plan validation, brachytherapy dose extraction, and independent QA processes.^19^, ^20^, ^21^ Building on these foundations, this work leverages ESAPI to enable seamless transfer of adapted plans from the Ethos CTgART system to the Halcyon, establishing the infrastructure necessary for a hybrid ART–IGRT treatment paradigm.

In this study, we developed and validated an end‐to‐end automated workflow that supports variable ART frequency by integrating adaptive and non‐adaptive treatment systems. The framework automates key plan‐transfer steps, minimizes manual workload, and maintains dosimetric fidelity between adaptive and IGRT deliveries. Multi‐institutional tests at two large academic institutions further demonstrated the tool's generalizability, with site‐specific feedback informing refinements that led to successful deployment. In addition, an Ethos‐only IGRT workflow was developed to support centers without access to a Halcyon, allowing adapted plans generated on Ethos to be subsequently delivered as IGRT treatments on the same platform. Together, these results establish a scalable, vendor‐aligned automation framework that improves clinical flexibility, optimizes machine utilization, and promotes broader adoption of ART workflows across diverse practice settings.

## 2 METHODS

### 2.1 Adapt‐on‐demand concept

We developed an end‐to‐end clinical workflow to leverage the adaptive planning strengths of the Varian Ethos v2.0 platform (equipped with HyperSight CBCT) alongside the high‐throughput IGRT delivery capabilities of the Varian Halcyon. This framework enables dynamic switching between adaptive and standard IGRT workflows, accommodating patient‐specific anatomical changes without committing to daily ART.

Patients are initially planned using Ethos to enable future adaptive treatments. The reference plan is imported into ARIA and configured as an IGRT plan for Halcyon delivery. Treatment typically begins on Halcyon, allowing physicians to review CBCT images offline in ARIA and trigger adaptation when clinically indicated. When adaptation is requested, treatment shifts to Ethos. Adapted plans are then converted and imported back into ARIA to allow continued IGRT delivery on Halcyon. This workflow reduces the ART resource burden while broadening adaptive access across a wider patient population.

### 2.2 Automation via ESAPI

**FIGURE 1:**
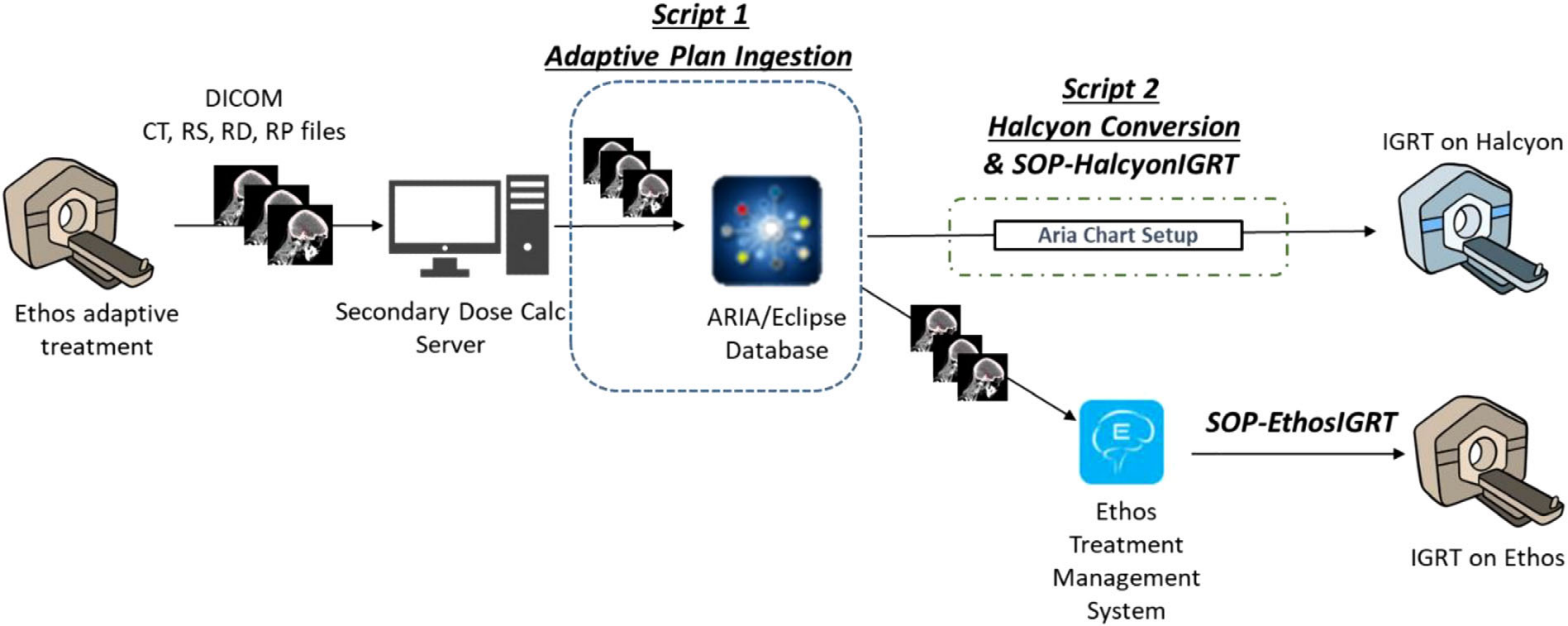
Automation pipeline for enabling adapted plan delivery on Halcyon and Ethos IGRT platforms. The Ethos TPMS automatically exports DICOM files to a pre‐configured network directory upon plan approval. The Plan Ingestion script monitors this directory via a file watcher, identifies approved adaptive plans by parsing DICOM metadata, and facilitates import into the ARIA/Eclipse database via DICOM C‐STORE. The Halcyon Conversion script then performs downstream processing including chart setup, dose grid reset, material table application, and plan regeneration using ESAPI. An alternative pathway supports IGRT delivery on Ethos via re‐import into the Ethos TPMS.

Figure 1 illustrates the technical steps required to enable IGRT treatment on both the Halcyon and Ethos platforms. The process begins with a standard feature of the Ethos system: upon completion of an adaptive session—including both technical and clinical plan approval within the Ethos TPMS—DICOM files (including CT images, structures, plans, and dose data) from both the adapted and scheduled plans are automatically exported to a pre‐configured network directory. This export is the standard pathway used for secondary dose calculation verification; at our institution, an in‐house Monte Carlo dose engine is utilized for the secondary dose calculation.^22^

#### 2.2.1 Adaptive plan ingestion

Critically, the Ethos TPMS is a closed system that does not expose an API for external queries. To bridge this gap, we developed a file watcher that monitors the Ethos DICOM export directory. When new files appear, the script parses DICOM metadata (including Plan Intent and Approval Status tags) to identify approved adaptive plans and distinguish them from scheduled or reference plans. Upon identification, the script facilitates transfer of the DICOM files into the ARIA database via DICOM C‐STORE. This eliminates the previously manual and time‐consuming process—measured at approximately 20 min based on historical care path task timestamps—of manually exporting the plan from the Ethos TPMS, unzipping the files, sorting the DICOM components, and importing them into Eclipse.

Once the DICOM files are written into the ARIA database, two IGRT delivery options are available. The files can be re‐imported into the Ethos TPMS for IGRT delivery on the Ethos platform itself. This Ethos‐only pathway is particularly relevant for centers that do not have a separate Halcyon and represents a broadly applicable use case. Alternatively, the primary objective of this work is to establish a workflow and tools that enable IGRT delivery on the Halcyon platform, as described below.

#### 2.2.2 Halcyon conversion

**FIGURE 2:**
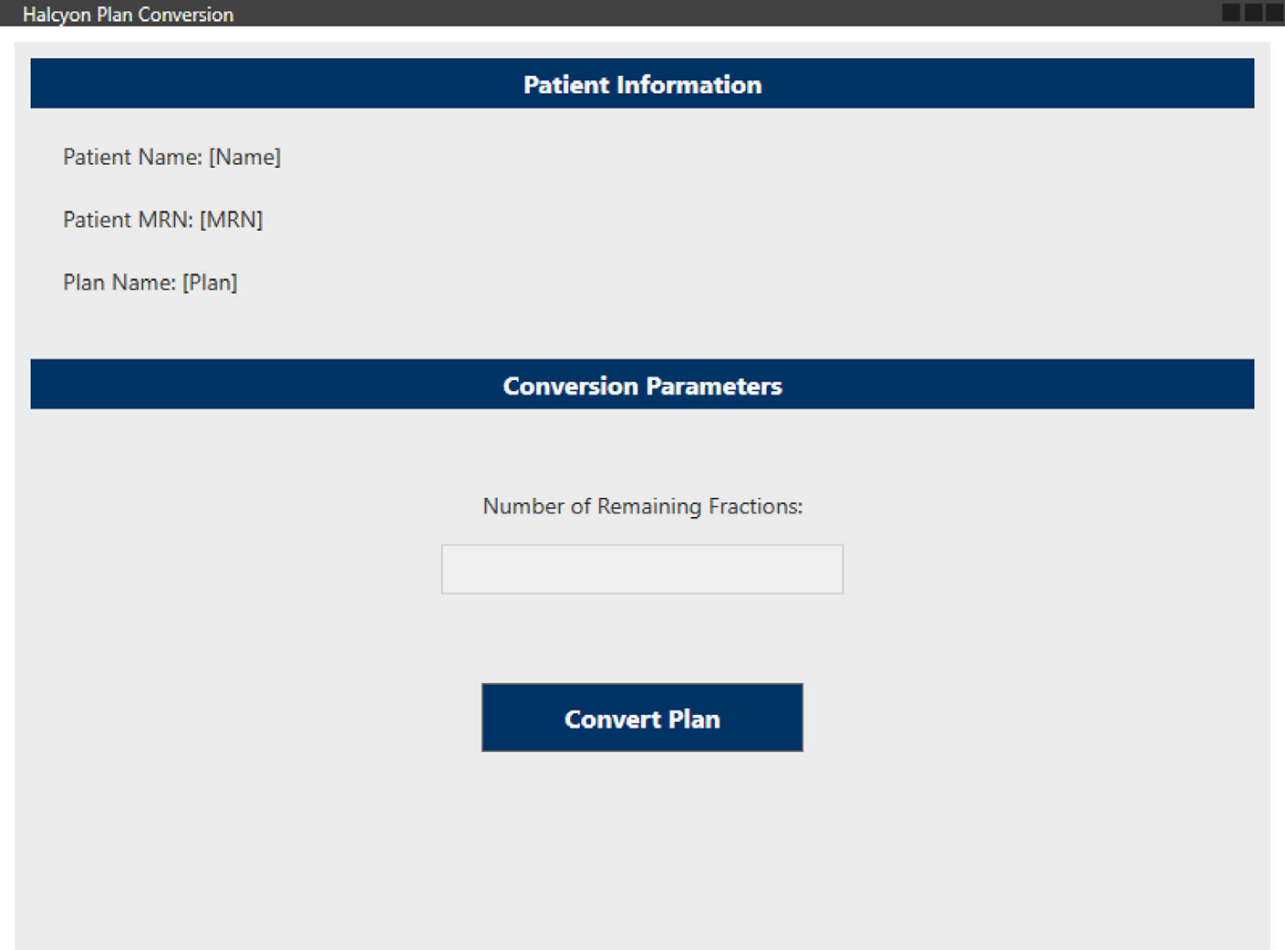
Graphical user interface for the Halcyon plan conversion module, showing the Plan Conversion tab with patient information, conversion parameters, and the Convert Plan action.

A second ESAPI script automates the preprocessing required to make the adapted plan compatible with Halcyon delivery. The script resets the dose calculation grid, inserts the standard Halcyon imaging field, and modifies prescription parameters to meet Halcyon delivery requirements. Although the beam geometries between Ethos and Halcyon remain identical, the module regenerates a Halcyon‐compatible RT Plan object that preserves all monitor units and beam definitions. A full dose recalculation is then triggered to ensure consistency, particularly in response to any changes in material assignments or imaging geometry. The script includes built‐in MU verification, comparing target versus actual MU per beam to within a configurable tolerance (default: 0.01 MU). Figure 2 displays the graphical user interface (GUI) for the Halcyon Conversion module.

As much of the ARIA chart setup as currently allowed by the available Eclipse API is automated by the second script. The remaining configuration steps—including prescription linkage, plan scheduling, and tolerance table assignment—must still be completed manually due to ESAPI v16.1 limitations. A detailed standard operating procedure (SOP) is provided as Supplementary Document S1 to guide these manual steps.

### 2.3 Evaluation

We conducted a six‐month prospective evaluation comparing 100 adapt‐on‐demand fractions processed through the automated workflow with 100 historical fractions processed manually. We compared the time to set up the IGRT treatment on Halcyon manually versus with the ESAPI scripts, measured via ARIA care path task timestamps. An unpaired two‐tailed *t*‐test (α = 0.05) was used to assess the statistical significance of the time reduction, as the manual and automated groups represent independent samples.

Throughout the evaluation, every ESAPI invocation generated a detailed log file capturing module‐level success or failure codes, error messages, and execution durations. These logs were reviewed to identify and resolve issues—such as database connectivity errors or DICOM import conflicts—and to quantify the robustness of the automation pipeline.

### 2.4 Scheduling impact

We analyzed 19 months of Ethos machine utilization data since machine go‐live, including a 6‐month period following the implementation of IGRT delivery on Halcyon. This analysis aimed to descriptively assess temporal trends in ART accessibility and overall machine efficiency coinciding with workflow changes. To quantify the effect, we tracked the percentage of ART versus IGRT fractions delivered on Ethos, using this ratio as an indicator of adaptive treatment utilization. Additionally, we evaluated daily machine throughput by measuring the time allocated to ART and IGRT, based on scheduled treatment slot durations from the ARIA scheduling system, before and after the workflow change. We note that multiple factors beyond the automation tool—including physician learning curves, evolving institutional protocols, and patient case mix—may have influenced these trends.

### 2.5 Multi‐site scalability

To improve versatility of adapt‐on‐demand workflows, we collaborated with two large academic centers. The primary goal was to enhance flexibility and usability—particularly in settings without dedicated in‐house programming support.

Because both partner institutions employ the commercial secondary dose calculation software Mobius (Varian Medical Systems, Palo Alto, CA) integrated with their treatment systems, workflow modifications were required. A Mobius‐compatible version of the script was developed, allowing site‐specific configuration of verification systems and process checkpoints. The implementation package included installation documentation and example datasets to support local setup and validation.

## 3 RESULTS

### 3.1 Halcyon conversion workflow

Table 1 summarizes the technical setup required to prepare an Ethos‐adapted plan for IGRT delivery on the Halcyon platform, along with the automation capabilities supported by Eclipse ESAPI v16.1. Certain steps cannot be fully automated due to interoperability limitations between the Ethos TPMS and Eclipse treatment planning systems. For example, material definitions differ between the two systems—such as the naming convention for titanium—which requires manual selection of the appropriate material table to ensure compatibility and accurate dose calculation in Eclipse. Additionally, Eclipse occasionally flags minor discrepancies in MLC positions, often requiring submillimeter adjustments that must be resolved manually.

**TABLE 1:** Technical steps for preparing an Ethos‐adapted plan for Halcyon IGRT delivery, with corresponding automation support via Eclipse ESAPI v16.1. Non‐automatable tasks reflect current API or system compatibility limitations. See Supplementary Document S1 for setup guidance.

Process step	Mandatory/optional	ESAPI V16.1
Insert kV CBCT field	Mandatory	✓
Change machine name	Mandatory	✓
Reset MLC position	Mandatory, only when Eclipse occasionally flags the MLC position is not compatible for Halcyon delivery upon plan approval	N/A
Reset reference point for dose accumulation	Optional, per user workflow	N/A
Recalculate dose	Mandatory, when resetting reference point or resetting MLC position	✓
Setup default calculation models	Mandatory	✓
Select physical material table	Mandatory, when dose recalculation is needed and AXB is selected	N/A
Revise number of treatment fractions	Optional, per user workflow	✓
Link plan to prescription	Mandatory	N/A
Approve plan	Mandatory	N/A
Schedule plan	Mandatory	N/A

Abbreviations: AXB, Acuros XB algorithm; ESAPI, Eclipse Scripting API; kV CBCT, kilovoltage cone‐beam computed tomography; MLC, multileaf collimator.

Furthermore, ARIA chart setup steps are not supported by ESAPI v16.1, and these configurations tend to vary significantly between institutions. To aid reproducibility, a SOP is provided as Supplementary Document S1 for reference, and the ESAPI automation scripts and configuration templates used in this work are available from the corresponding author upon reasonable request.

### 3.2 Efficiency evaluation

Analyzing the time savings due to automation, the mean conversion time decreased from 45 ± 8.5 min under the historical manual workflow to 8 ± 2.3 min with the automated pipeline (*p* < 0.001, unpaired *t*‐test).

To verify plan conversion fidelity, we performed a direct comparison of MLC leaf positions between the Ethos‐native adaptive plans and their Halcyon‐converted counterparts. Across all evaluated cases, MLC leaf positions were identical at every control point, confirming that the conversion process preserves the beam geometry exactly. This is expected by design: the Ethos and Halcyon share identical MLC hardware, and the conversion script modifies only the machine identifier, imaging field, and dose calculation grid without altering any beam‐defining parameters.

### 3.3 Observed scheduling trends

**FIGURE 3:**
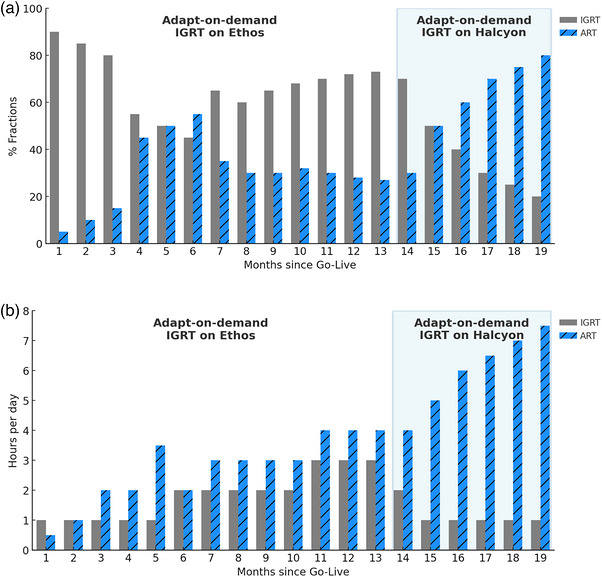
Observed trends in ART utilization and machine scheduling following workflow changes. (a) Monthly percentage of ART and IGRT fractions delivered on Ethos before and after the transition of IGRT to Halcyon. (b) Average hours per day dedicated to ART and IGRT on the Ethos system over the same period, based on scheduled treatment slot durations from the ARIA scheduling system. Multiple factors beyond the automation tool may have contributed to these trends (see Discussion).

Figure 3a illustrates the monthly trend in the proportion of ART and IGRT fractions delivered on the Ethos system since go‐live. Initially, the institution operated under the default configuration, providing both daily ART and IGRT on Ethos, with ART fractions comprising only 6% to 17% of total treatments. Following the implementation of the adapt‐on‐demand workflow with IGRT support on Ethos (months 8–12), ART utilization increased to 46%. However, IGRT cases delivered on Ethos also grew during this period, and by month 13, the proportion of ART had decreased to 27% as IGRT cases consumed a significant share of available machine time.

To address this, IGRT delivery was transitioned to Halcyon starting in month 14. This change coincided with a sharp decline in IGRT use on Ethos and a substantial increase in ART utilization, which reached 77% of total Ethos treatments by month 18. We note that these trends were likely influenced by multiple factors, including increasing physician familiarity with the adaptive workflow, evolving institutional protocols, and changes in patient case mix, in addition to the workflow automation itself.

Figure 3b further illustrates this trend in terms of machine time: after the transition, daily ART usage on Ethos expanded to over 7 h per day, while IGRT time dropped to less than 1 h. This shift provided greater flexibility in scheduling ART sessions around physician availability, improving both machine efficiency and adaptive access.

### 3.4 Expansion to external institutions

To broaden accessibility, we developed a Mobius‐compatible version of the Plan Ingestion tool for general clinical use. The updated application integrates three main functions—Import, Plan Conversion, and Configuration—within a single, user‐friendly interface. Users can select an export directory from the adaptive platform, where the tool automatically identifies approved adaptive‐plan DICOM sets, labels them by patient and fraction, and facilitates transfer to ARIA via DICOM C‐STORE.

To accommodate different clinical environments, we added configurable options that allow users to define their verification systems and specify workflow checkpoints aligned with their local practice. The release package includes detailed installation guides, example datasets, and a GUI designed to minimize technical barriers for users without programming experience.

Feedback from both partner institutions guided further refinement of the tool—improving error handling, user notifications, and SOP documentation. After iterative testing and validation, both partners confirmed the tool functioned as intended and was ready for clinical deployment.

These enhancements established a flexible and scalable framework that supports implementation across centers using CTgART systems and Halcyon linacs, regardless of their IT or physics support infrastructure.

## 4 DISCUSSION

This work presents a practical framework that enhances the flexibility of CTgART. By integrating adaptive and non‐adaptive workflows, the developed automation enables treatment teams to tailor adaptation frequency to patient‐specific needs, rather than committing to a rigid daily schedule. This flexibility allows centers to maximize adaptive resources, improve machine utilization, and expand access to ART without compromising treatment quality.

At our institution, implementing this integrated Ethos–Halcyon workflow coincided with a substantial increase in ART utilization. As non‐adaptive IGRT fractions were transitioned to Halcyon, Ethos adaptive capacity expanded, and overall ART time rose from approximately 4 h per day to nearly 8 h per day. The resulting increase in available adaptive slots alleviated scheduling bottlenecks, allowing physicians to align treatment times more easily with their clinical availability. While we cannot attribute these changes solely to the automation tool—given concurrent changes in physician experience, institutional protocols, and patient demographics—the temporal association supports the potential for coordinated dual‐platform use to enhance ART throughput and clinical accessibility.

The automation framework also provided a significant gain in operational efficiency, reducing the manual setup time for adapted plan transfer from roughly 45 to 8 min. This reduction not only streamlined the clinical workflow but also minimized the likelihood of manual error, improved staff utilization, and supported more predictable daily scheduling. Collectively, these time savings translate into greater adaptive capacity within existing clinical resources, reinforcing the practical value of automation in routine ART operations.

A key consideration in the adapt‐on‐demand paradigm is how physicians decide when to trigger adaptation. In our workflow, this decision framework was intentionally designed to preserve the flexibility of Ethos rather than replace online adaptation. Physicians retain full access to on‐unit adaptive planning whenever clinically appropriate. In practice, we operate through two primary pathways. The first is a pre‐scheduled adaptive frequency (e.g., weekly or at predefined treatment milestones), allowing physicians to perform online adaptation with full real‐time dosimetric evaluation during the session. The second is a physician‐initiated offline review pathway. Patients treated on Halcyon undergo daily CBCT imaging, which physicians review in ARIA. Adaptation is triggered based on clinically meaningful anatomical changes—such as tumor regression, organ filling variation, or significant weight loss. When needed, we provide CBCT‐based plan recalculation to generate quantitative dose comparisons to support decision‐making. However, a key lesson from implementation is that physician clinical judgment frequently outweighs rigid metric‐based triggers; therefore, the final decision to adapt remains physician‐driven. In addition, a safety layer exists in which substantial geometric deviations (e.g., ≥2 cm shift or notable SSD change) identified by the therapy team automatically prompt dose recalculation for physician review. Regardless of which pathway is chosen, the physician is always presented with both the scheduled and adapted plan comparisons during the online adaptive process; it is the trigger mechanism that varies—whether pre‐scheduled, physician‐initiated, or RTT‐initiated. Ultimately, adaptation frequency reflects a balance between clinical relevance and operational feasibility, with approximately half of adaptations arising from pre‐scheduled sessions and half from physician‐ or RTT‐triggered evaluation.

Importantly, the introduction of new‐generation CBCT technology (HyperSight, Varian Medical Systems) provides distortion‐free, HU‐accurate imaging that supports direct dose calculation, enabling a streamlined simulation‐omitted (direct‐to‐unit) workflow.^24^, ^25^, ^26^, ^27^, ^28^, ^29^, ^30^, ^31^ Our automation framework integrates naturally with this capability—allowing patients to undergo adaptation at the first fraction, generate a clinically deliverable plan, and then continue subsequent fractions as IGRT on Halcyon. This hybrid model not only reduces the need to commit all treatments to the adaptive platform but also frees additional adaptive slots, expands access to simulation‐omitted processes, and enables patients to begin treatment earlier while maintaining dosimetric precision and workflow efficiency.

To promote reproducibility and scalability, a detailed SOP for Halcyon IGRT delivery is provided as Supplementary Document S1. This resource offers step‐by‐step implementation guidance adaptable to varying institutional infrastructures, including those without dedicated programming support. The ESAPI source code and associated configuration files for the Plan Ingestion and Halcyon Conversion modules are available from the corresponding author upon reasonable request and subject to institutional and vendor data‐sharing policies.

### 4.1 Limitations

Several limitations of this work should be acknowledged. First, the scheduling and utilization data were derived from a single institution. While external validation at two collaborating centers confirmed that the workflow could be successfully integrated across diverse environments, utilization trends at these sites were not formally analyzed. Future work will focus on assessing utilization metrics and long‐term clinical impact at these partner sites.

Second, the current automation primarily supports the Ethos‐to‐IGRT direction. Transitioning a patient back from IGRT to Ethos for a new adaptive session requires re‐enrollment in the Ethos TPMS, which involves manual steps outside the scope of the current ESAPI framework. This unidirectional limitation reflects the closed nature of the Ethos TPMS, which does not currently expose an API for external plan import or session creation. Bidirectional automation remains an area for future development pending vendor‐side interoperability improvements.

Third, adaptation triggers in the current workflow are based on qualitative physician assessment of offline CBCT images, without the on‐unit dosimetric evaluation that would be available during a full Ethos adaptive session. While this approach is practical and efficient, it may miss dosimetrically significant changes that are not visually apparent.

Fourth, ESAPI v16.1 does not support full automation of ARIA chart setup (e.g., prescription linkage, plan scheduling, tolerance table assignment), requiring residual manual steps that vary by institution. These steps are documented in Supplementary Document S1 but represent a barrier to fully hands‐free operation.

Finally, the observed utilization trends were likely influenced by multiple confounding factors—including evolving physician comfort with ART, changes in patient case mix, and institutional policy adjustments—that cannot be isolated from the effect of the automation tool itself. A controlled, multi‐institutional study would be needed to rigorously quantify the tool's independent contribution to adaptive utilization.

### 4.2 Future directions

Beyond immediate implementation, this solution establishes a foundation for adaptive therapy on demand—a model balancing precision with practicality. The framework also lays the groundwork for integrating artificial intelligence and automation to further streamline image analysis, plan adaptation, and decision support. For instance, deep‐learning dose prediction models have demonstrated the ability to derive optimization goals leading to high‐quality offline replans.^23^ Likewise, one‐stop automation frameworks have been trialed that illustrate that imaging, planning, and delivery can be seamlessly orchestrated with minimal human intervention.^24^ Ultimately, these advancements make CTgART more flexible, scalable, and resource‐efficient, positioning it for broader adoption as a modern standard of care.

## 5 CONCLUSION

We developed and validated a scalable Adapt‐on‐Demand framework that enables flexible transitions between CTgART on the Varian Ethos platform and IGRT delivery on the Varian Halcyon, supported by ESAPI‐based workflow automation. The framework reduced plan conversion time from 45 to 8 min and was associated with a substantial increase in ART utilization at our institution. Successful deployment at two external academic centers without code modification confirmed the tool's portability and generalizability. An Ethos‐only IGRT pathway further extends applicability to centers without a separate Halcyon. By addressing the infrastructure gap between adaptive and non‐adaptive delivery systems, this work provides a practical and reproducible model for implementing variable‐frequency ART in routine clinical practice, with the goal of improving machine utilization, clinical throughput, and patient access to ART.

## AUTHOR CONTRIBUTIONS

Mu‐Han Lin, Taoran Li and Sean Domal designed the study. Sean Domal developed all scripts and user interfaces and drafted the first iteration of the manuscript. Mu‐Han Lin provided clinical supervision and input to the project. All authors revised and approved the final manuscript.

## FUNDING INFORMATION

This work was supported by a sponsored research agreement between Varian Medical Systems, Inc. and the Department of Radiation Oncology at UT Southwestern Medical Center.

## CONFLICT OF INTEREST STATEMENT

This project received research funding from Varian Medical Systems, Inc. No other conflicts of interest are reported by the authors specific to this work.

## AI USAGE DECLARATION

During the preparation of this work the author(s) used ChatGPT in order to curate the manuscript. After using this tool/service, the author(s) reviewed and edited the content as needed and take(s) full responsibility for the content of the published article.

## Supporting information




**Supporting Information**: acm270612‐sup‐0001‐SOP.pdf

## Data Availability

ESAPI automation scripts and associated configuration files used for plan ingestion and Halcyon conversion are available from the corresponding author upon reasonable request.
