# Supplementary material for: Adapt‐on‐demand: Enabling flexible and scalable adaptive radiotherapy through workflow automation
Source: J Appl Clin Med Phys. 2026 May 13;27(5):e70612. doi: 10.1002/acm2.70612 (PMC13172267; doi:10.1002/acm2.70612)
Supplement: Supplementary file 1 — Supporting Information: acm270612‐sup‐0001‐SOP.pdf [file ACM2-27-e70612-s001.pdf]

SOP

Standard Operating Procedure

Script Driven Ethos Adapted Plan (ADP) to Halcyon Conversion Process

Instructions for Use:

This Standard Operating Procedure (SOP) outlines the workflow and methodology for converting Ethos-adapted treatment plans into deliverable plans for the Halcyon system via script. The document provides step-by-step guidance on application configuration, script driven plan import, CT isocenter verification, script driven adapted plan conversion, prescription verification and plan scheduling, and additional discussion, aiming to standardize the procedure and enhance efficiency while maintaining treatment accuracy and patient safety.

Disclaimer: This document is intended for use by trained clinical staff and radiation oncology professionals. While the described workflow has been rigorously tested and validated, the authors and institution assume no liability for any errors, omissions, or outcomes resulting from the use of this SOP. Users are responsible for verifying all parameters, quality assurance results, and adherence to institutional and regulatory guidelines prior to clinical implementation.

Table of Contents

DICOM Daemon Setup 3
Application Configuration 10
Automated Adaptive Plan Ingestion 13
Semi-Automated Adaptive Plan Conversion 19
CT Isocenter Verification 29
Prescription Association and Plan Scheduling 30
Plan Preparation and Plan Retirement 35
Additional Notes 38

DICOM Daemon Setup

Purpose: To provide step-by-step instructions for setting up the Varian DICOM DB Daemon to programmatically extract DICOM radiotherapy data from the Aria database.

Key Concepts: Senders vs. Listeners

Before diving into configuration, it's essential to understand how DICOM network communication works. This will help you understand why certain settings are required.

DICOM Network Roles

In DICOM networking, there are two fundamental roles that applications can play:

Listener (SCP - Service Class Provider)

- Waits passively on a specific port for incoming connections
- "Provides" a service when another application connects and requests it
- **Must have a listening port configured** because other systems need to know where to connect
- Think of it like a phone - it sits waiting for calls to come in

Sender/Caller (SCU - Service Class User)

- Actively initiates connections to other systems
- "Uses" services provided by SCPs (listeners)
- **Does NOT need its own listening port** - it makes outgoing connections only
- However, it must be listed as "trusted" by any listener it wants to connect to
- Think of it like making a phone call - you dial out to someone else

Component Roles in This Setup

Here's how each component in our system functions:

Component	Role	Needs Port?	What It Does
**DB Daemon**	SCP (Listener)	**YES**	Listens for C-MOVE requests, then pushes data to File Daemon
**File Daemon**	SCP (Listener)	**YES**	Listens for incoming DICOM files and saves them to disk
**DCMTK**	SCU (Sender)	**NO**	Sends C-MOVE command; does NOT receive files directly

Data Flow Diagram

The following diagram shows how data flows through the system:

DATA FLOW

Step 1: ESAPI Script runs DCMTK command

↓

DCMTK sends C-MOVE request to **DB Daemon**
("Please send Plan XYZ to File Daemon")

↓

Step 2: DB Daemon retrieves data from Aria

↓

DB Daemon sends DICOM files to **File Daemon**
(RT Plan, Structure Set, CT Images, Dose)

↓

Step 3: File Daemon saves files to disk
Output: `C:\Temp\FileService\VMSFD\[PatientID]\`

Understanding "Trusted" AE Titles

Each listener (SCP) maintains a list of "trusted" applications that are allowed to connect to it. Think of this as an access control list. When you add a trusted AE title, you're telling the listener: "If someone connects claiming to be this AE title from this IP address, allow them in."

Required Trust Relationships:

Listener	Must Trust	Why
**DB Daemon**	DCMTK	DCMTK sends C-MOVE commands
**DB Daemon**	File Daemon	DB Daemon needs to know destination
**File Daemon**	DB Daemon	File Daemon receives files from DB Daemon

Prerequisites

Before beginning the setup, ensure you have the following:

- A Windows computer that is a Varian client connected to the Aria database
- Administrative access to install software and configure services
- Eclipse TPS installed (version 11 or later for ESAPI support)
- Contact information for your local Varian service representative
- Network information: IP addresses and available ports

Part 1: Install the DICOM DB Daemon

The DICOM DB Daemon must be installed by Varian personnel. This component provides access to DICOM-RT information stored in the Aria database.

Steps:

1. **Contact your local Varian service representative** and request installation of the Varian DICOM DB Daemon.
2. Specify which computer should host the daemon (server recommended, but workstation is acceptable for testing).
3. Ensure the target computer is a Varian client that can connect to the Aria database.
4. Wait for the service representative to complete the installation.

Verify Installation

After installation, you should see the following menu items in the Varian program group:

- DB Daemon Configuration
- File Daemon Configuration
- DICOM Daemon Service Configuration Wizard

Part 2: Install DCMTK

DCMTK (DICOM Toolkit) is an open-source toolkit for DICOM. We use it to send commands to the DB Daemon. Remember: DCMTK acts as a sender (SCU), so it does not need a listening port.

Steps:

1. **Download DCMTK** from: https://dicom.offis.de/dcmtk.php.en
2. Download the Windows binary package (e.g., dcmtk-3.6.x-win32-i386.zip).
3. Extract the ZIP file to a directory of your choice.

   Recommended: `C:\variandeveloper\tools\dcmtk-3.6.0-win32-i386`
4. Verify movescu.exe and echoscu.exe exist in the bin folder.

TIP: Make note of the full path to the bin directory - you will need this later when configuring the ESAPI script.

Part 3: Configure the DB Daemon (Listener)

The DB Daemon is a listener (SCP) that must be configured with a listening port and a list of trusted applications.

Recommended AE Title Configuration

Component	AE Title
DICOM DB Daemon	VMSDBD1
DCMTK (your scripting tool)	DCMTK
VMS File Daemon	VMSFD

Steps:

1. Open Configuration Wizard
   - **Open Windows Start menu and navigate to the Varian program group.**
   - **Click "DB Daemon Configuration."**

2. Add New Service (Configure the Listener)
   - **Click "Add New Service."**
   - **Enter the DB Daemon's settings:**
   - **AE Title: VMSDBD1 (this listener's identity)**
   - **Port: 5678 (port this listener monitors)**
   - **Click "Next."**

3. Configure Database Connection
   - **Verify the Aria database connection settings and click "Next."**

4. Add Trusted AE Titles (Who Can Connect)
   - **Add entries for applications that will connect to this listener:**

   Add DCMTK (the sender that will request data):
   - **Click "Add" and enter:**
   - **AE Title: DCMTK**
   - **IP Address: IP of Eclipse/DCMTK computer**
   - **Port: 11112 (informational - DCMTK doesn't actually listen)**

   Add File Daemon (where data will be sent):
   - **Click "Add" again and enter:**

- **AE Title: VMSFD**
- **IP Address: IP of File Daemon computer**
- **Port: 5680 (MUST match File Daemon's listening port!)**

TIP: If all components are on the same computer, use 127.0.0.1 or the same IP for all entries.

5. Step 5: Complete Configuration
   - **Click "Next," configure service startup options, and click "Finish."**
   - **The DB Daemon service should now start running.**

Part 4: Configure the File Daemon (Listener)

The File Daemon is also a listener (SCP). It receives DICOM files from the DB Daemon and saves them to disk.

Steps:

1. Open File Daemon Configuration
   - **Click "File Daemon Configuration" from the Varian program group.**
   - **Click "Add New Service."**
2. Configure Service Settings
   - **Enter the File Daemon's settings:**
   - **AE Title: VMSFD (must match DB Daemon config)**
   - **Port: 5680 (must match DB Daemon config)**
3. Configure Output Directory
   - **Specify where DICOM files will be saved:**
   - **Example: C:\Temp\FileService\VMSFD**
   - **Ensure the directory exists with write permissions.**
4. Add Trusted AE Titles
   - **The File Daemon must trust the DB Daemon (which sends files to it):**
   - **AE Title: VMSDBD1**
   - **IP Address: IP of DB Daemon server**
   - **Port: 5678**
5. Complete Configuration
   - **Click "Next," configure service startup, and click "Finish."**

Part 5: Verify Services Are Running

- Open Windows Services (services.msc)
- Locate "Varian DICOM DB Daemon" and "Varian DICOM File Daemon"
- Verify both show "Running" status
- If not running, right-click and select "Start"

WARNING: If services fail to start, check Windows Event Viewer for error messages.

Part 6: Example ESAPI Script

Download the example Script

- Go to: https://github.com/VarianAPIs/Varian-Code-Samples
- Download: `Eclipse Scripting API/plugins/GetDicomCollection.cs`

Configure the Script

Edit the configuration constants to match your environment:

```
public const string DCMTK_BIN_PATH = @"C:\variandeveloper\tools\dcmtk-3.6.0-win32-
i386\bin";
public const string AET = @"DCMTK";           // Your AE title (sender)
public const string AEC = @"VMSDBD1";         // DB Daemon's AE title (listener)
public const string AEM = @"VMSFD";           // File Daemon's AE title
(destination)
public const string IP_PORT = @"192.168.15.1 5678"; // DB Daemon's IP and listening
port
```

WARNING: AE titles and ports must exactly match your daemon configurations. They are case-sensitive!

Run the Script

- Open Eclipse and load a patient with a plan, structure set, CT data, and calculated dose
- Go to Tools > Scripts
- Select GetDicomCollection.cs and click "Run"
- Wait for completion (Notepad will show results)

Locate Exported Files

Files are saved to the File Daemon's output directory:

```
C:\Temp\FileService\VMSFD\[Patient ID]\
```

Troubleshooting

Connection Error

- Verify DB Daemon service is running
- Check IP address and port match DB Daemon's listening config
- Ensure firewall isn't blocking the port

Association Rejected

- Verify DCMTK is in DB Daemon's trusted list
- Check AE titles match exactly (case-sensitive)
- Confirm IP address in trusted entry is correct

No Files in Output Directory

- Verify File Daemon service is running
- Check File Daemon port matches DB Daemon's VMSFD entry
- Ensure DB Daemon is in File Daemon's trusted list

Additional Resources

- **Varian Code Samples:** https://github.com/VarianAPIs/Varian-Code-Samples/wiki
- **DCMTK Documentation:** https://support.dcmtk.org/docs/
- **DCMTK Download:** https://dicom.offis.de/dcmtk.php.en

Application Configuration

Purpose: To establish global application parameters for plan ingestion and conversion features. Please note you must have a Dicom Daemon setup prior to using this application.

Steps:

1. Launch the application in external beam module of eclipse and it will default you to the Config tab. Click the Help tab for setup instructions and guidance on what to input. The first block on information is specific to the dicom daemon you setup (please see that section in the SOP). The second block is specific to your institutional setup in Eclipse. This includes calculation specific features, machine name, imaging device name etc. “Workflow Preferences” has default values, including MU tolerance (for plan conversion) and a default import folder location that may be adjusted for user preference. Lastly, the storage location defaults to specific location, but the user can further adjust this to their needs. This is the location where the error log is subsequently kept. When you have all information input – click “Save Configuration”. Each time you open the application this information will by default appear in the “Config” tab. You may update or change any of this information and re-save at any time.

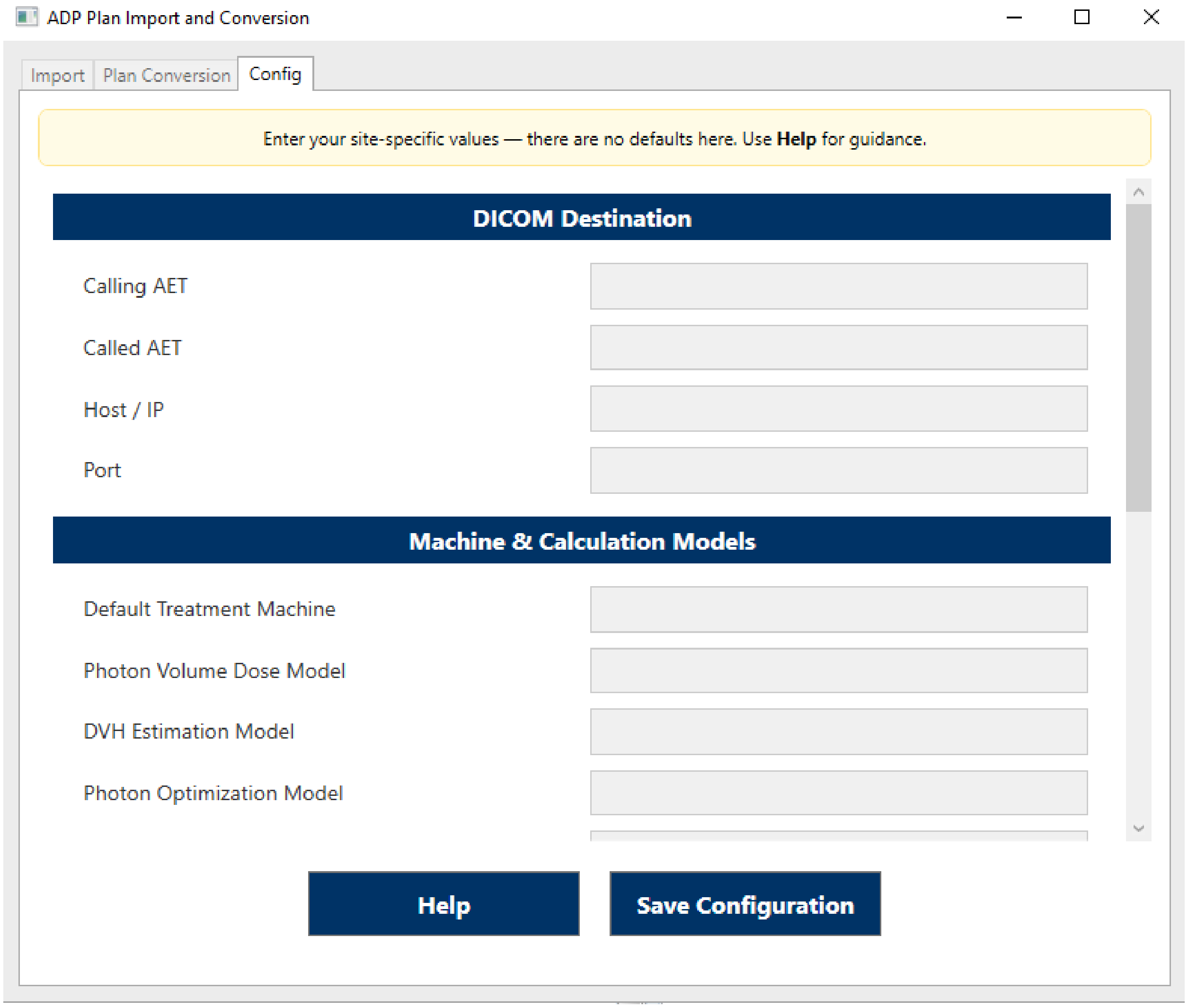

Eclipse Screen Capture – Config Tab

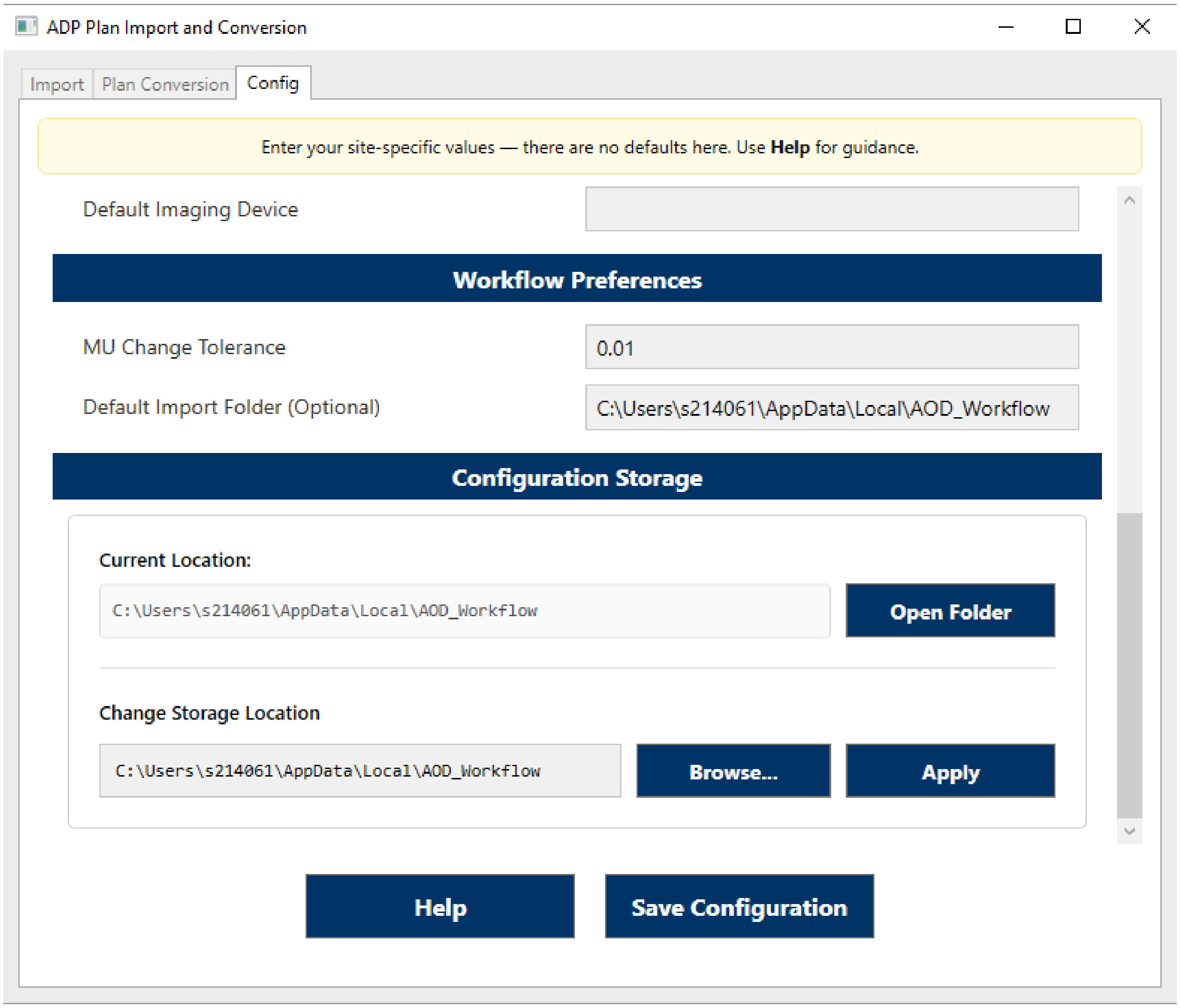

Eclipse Screen Capture – Config Tab

Automated Adaptive Plan Ingestion

Purpose: To ingest the adaptive plan into Eclipse for further Halcyon conversion.

Steps:

2. Launch GUI and navigate to Import tab – be sure you are in external beam planning module of Eclipse. Please note you must have the Configuration File created prior to navigating to this tab and attempting to ingest a plan.

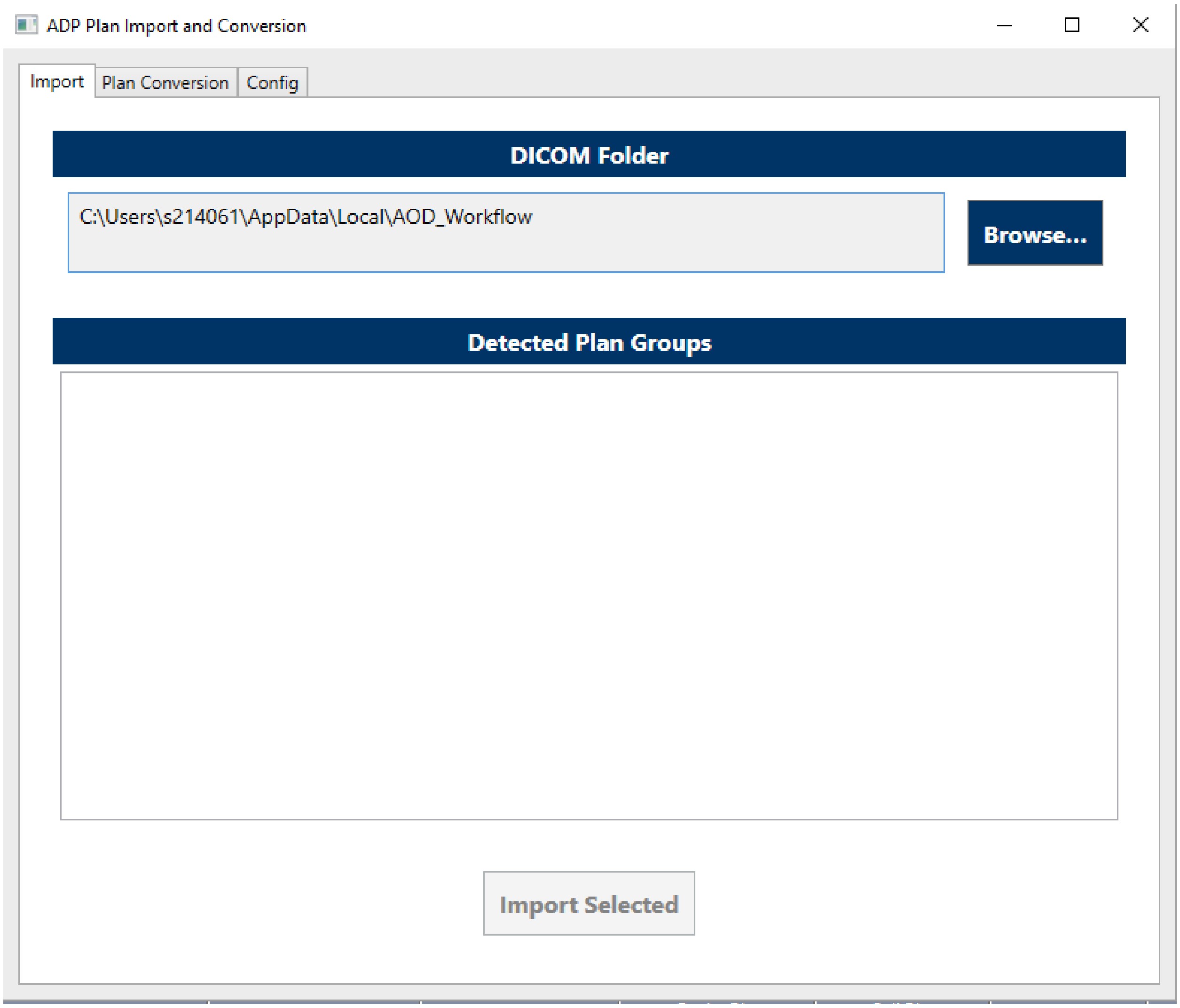

Eclipse Screen Capture – Plan Import Tab

3. Click “Browse button”. Navigate and click on the folder you wish the load and click “OK”.

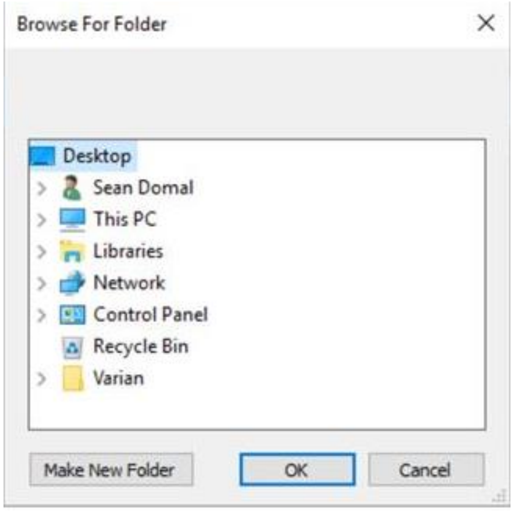

Eclipse Screen Capture – Browser Selection

4. The script will automatically load the folder and parse dicom files and group them into treatment sessions (images, structure set, plan, dose). Groups will be shown in the selection window.

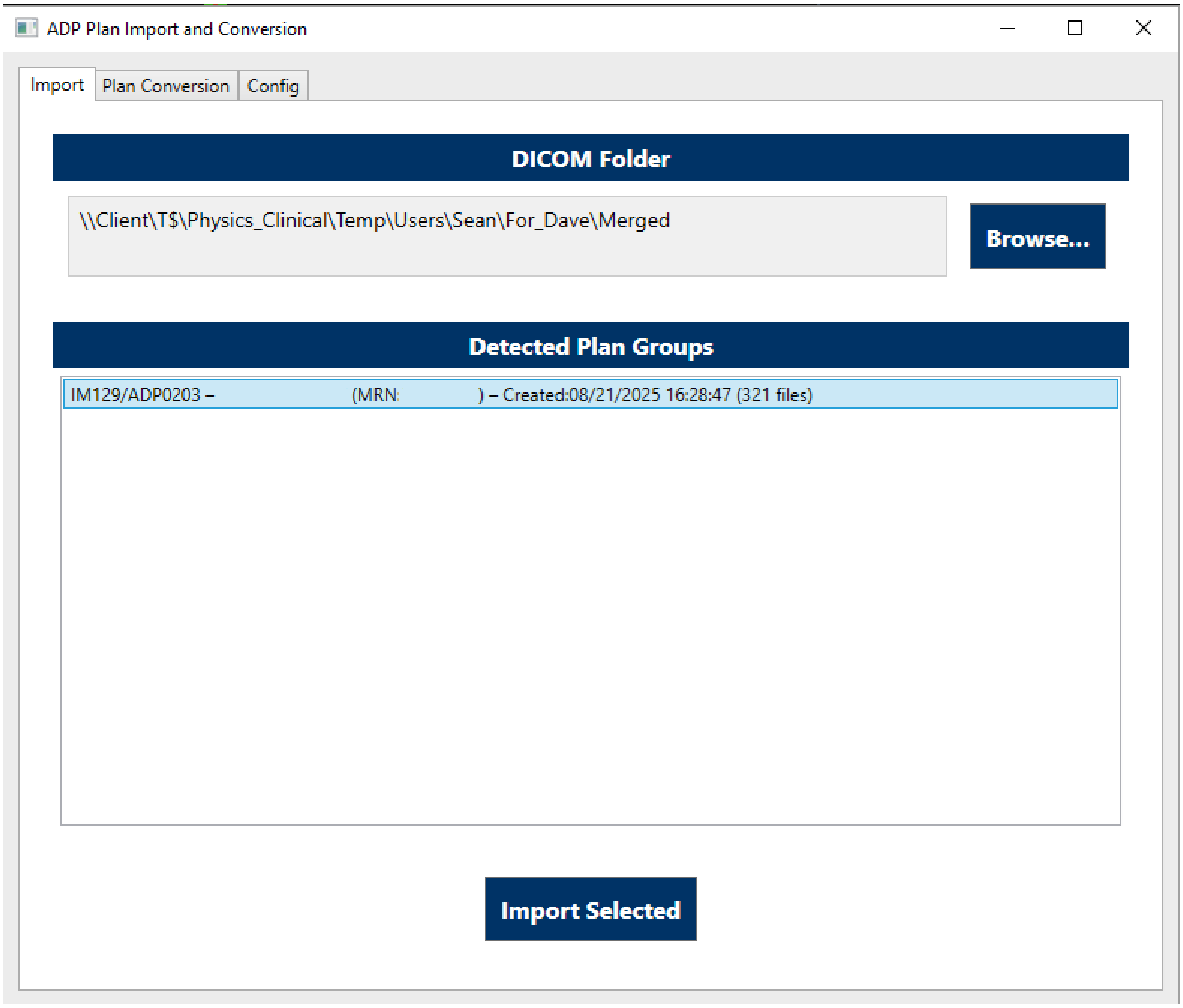

Eclipse Screen Capture – Selection Window

5. Select the treatment session you wish to ingest into Eclipse DB, and click “Import Selected”.

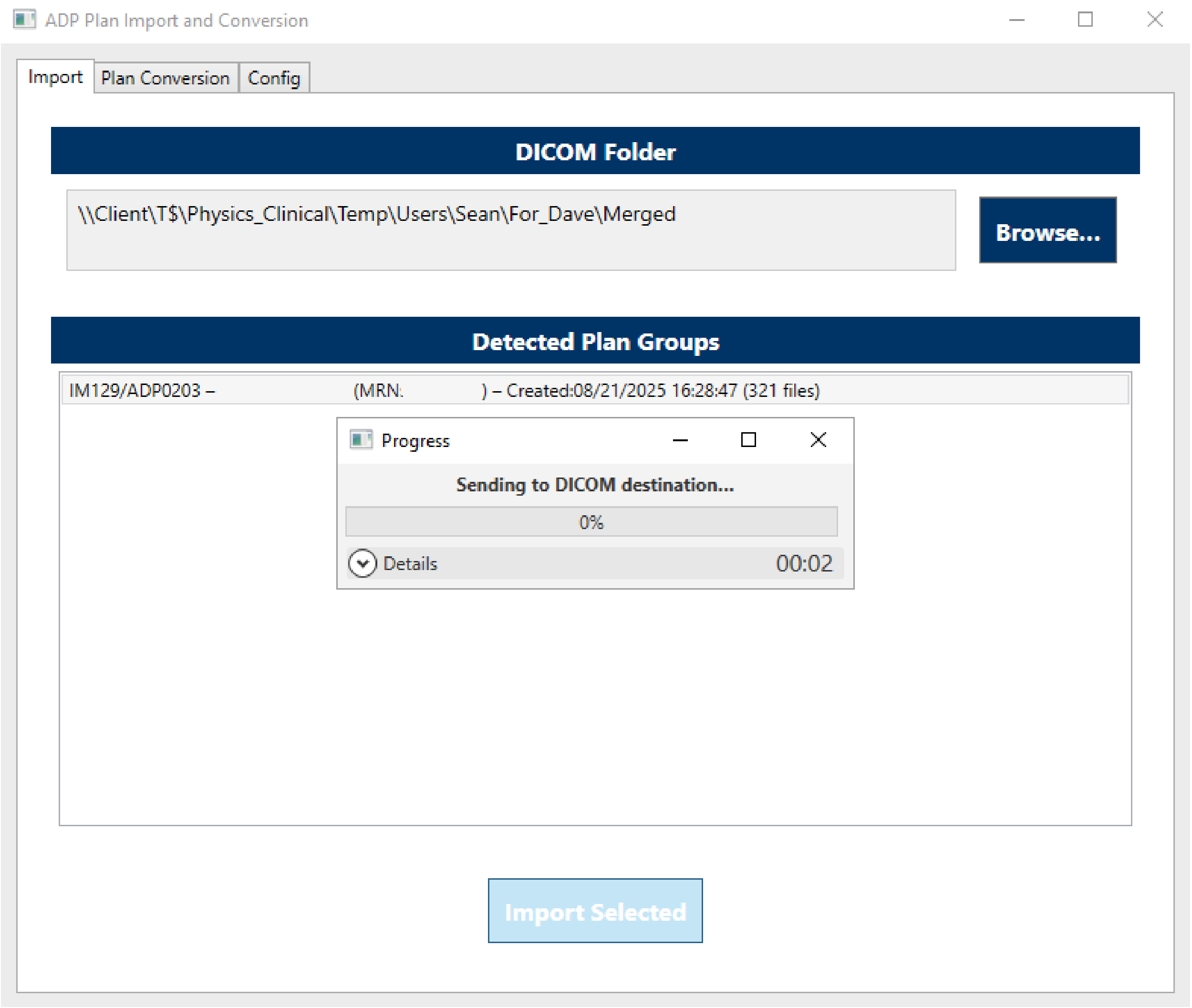

Eclipse Screen Capture – User Selection and Import

6. A Pop-up window and progress bar will update the user on the ingestion process. Once 100% has been reach close out of the progress bar and a “Success” popup will appear.

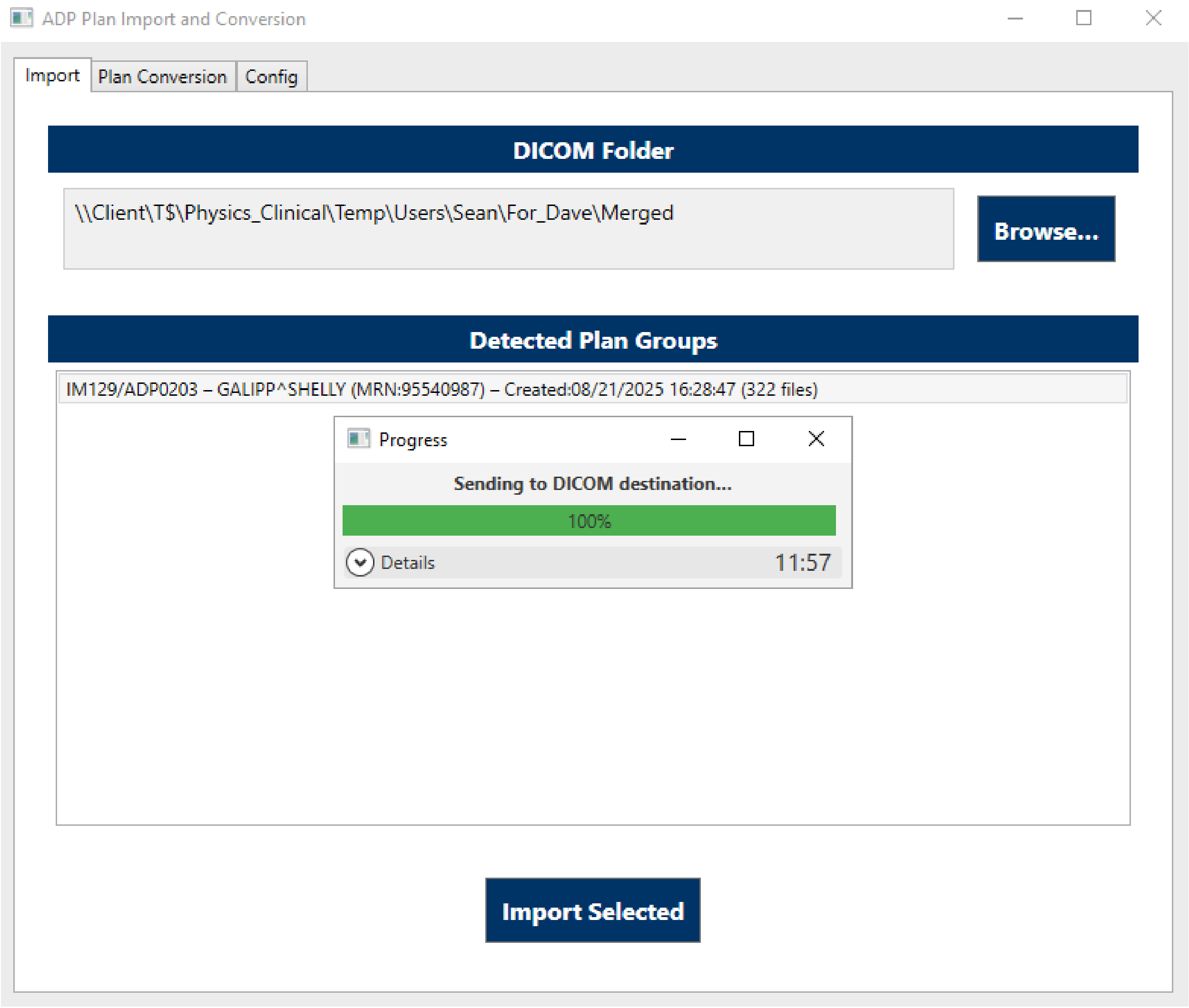

Eclipse Screen Capture – Import Completion

7. A pop-up will appear at conclusion of script process, the user must click “OK” and reload patient and course. The plan will be placed into the most recently created patient course.

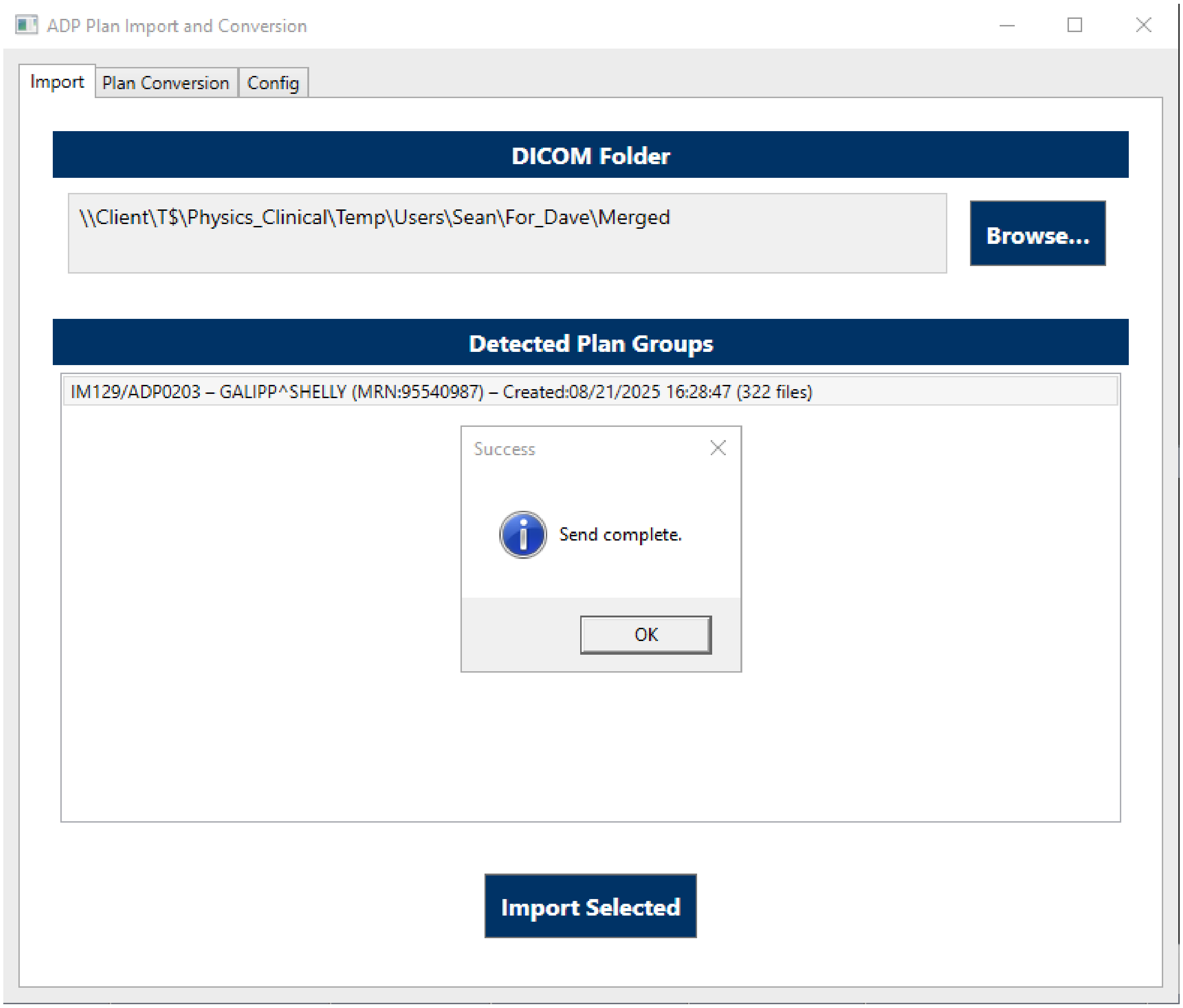

Eclipse Screen Capture – Close and Reload

*****Please note – you must close the application after plan ingestion and relaunch on the plan you want to convert for Halcyon delivery**

Semi-Automated Adaptive Plan Conversion

Purpose: <u>To aid in the conversion of ingested Ethos adapted plan to Halcyon deliverable plan.</u>

Steps:

1. Launch the script with the patient and adapted plan open in external beam planning. If you already have already filled out the Config tab and saved the configuration file – the GUI will default to the Plan Conversion tab. Immediately upon launching a pop-up will appear – you MUST reset dose, assign material table and assign CT number for any "Titanium" and SAVE before clicking "OK". The below steps walk through these items.

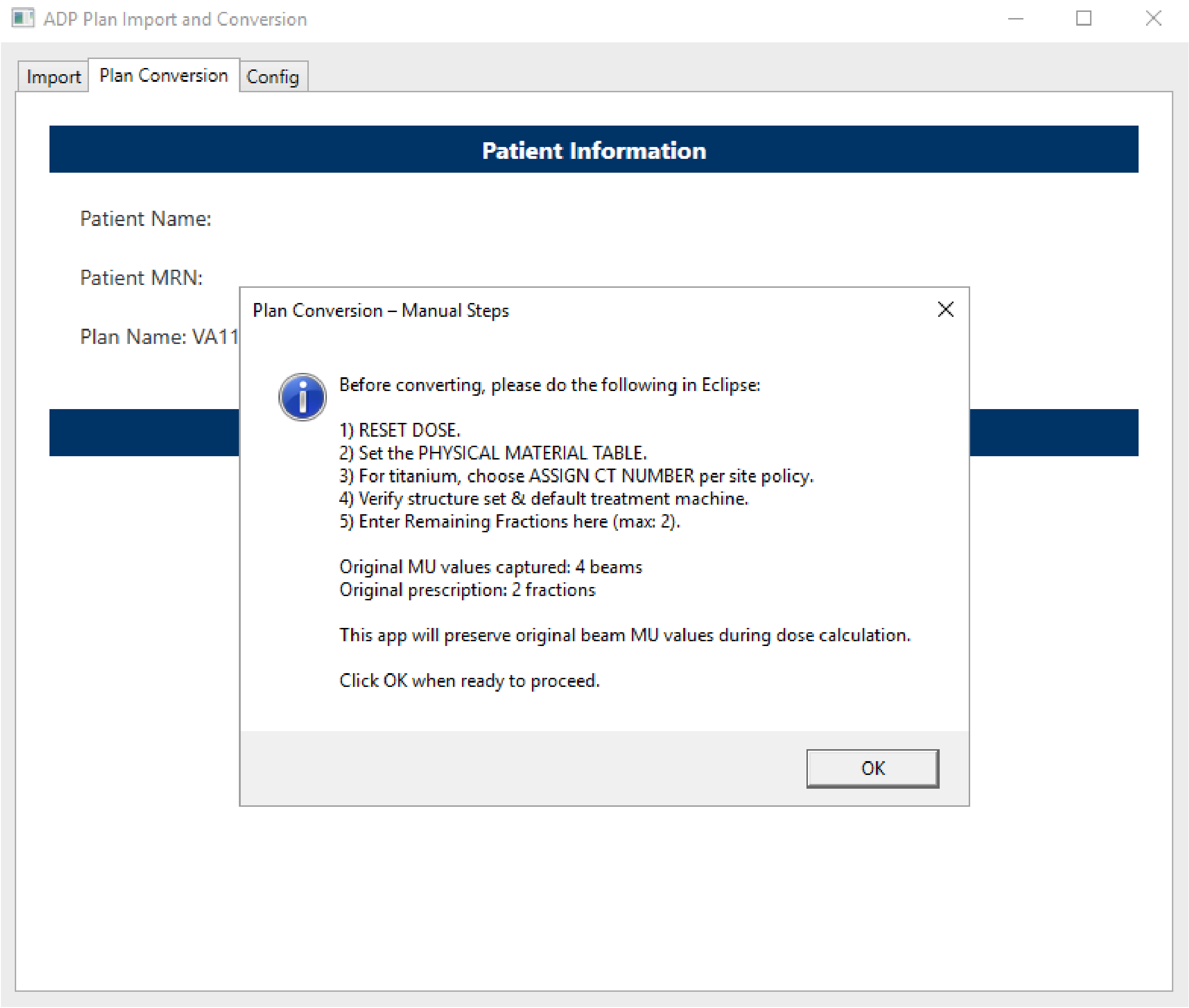

Eclipse Screen Capture – Plan Conversion Tab

2. Click “Reset Calculation Volume”. DO NOT close out of the Manual Setup Required popup.

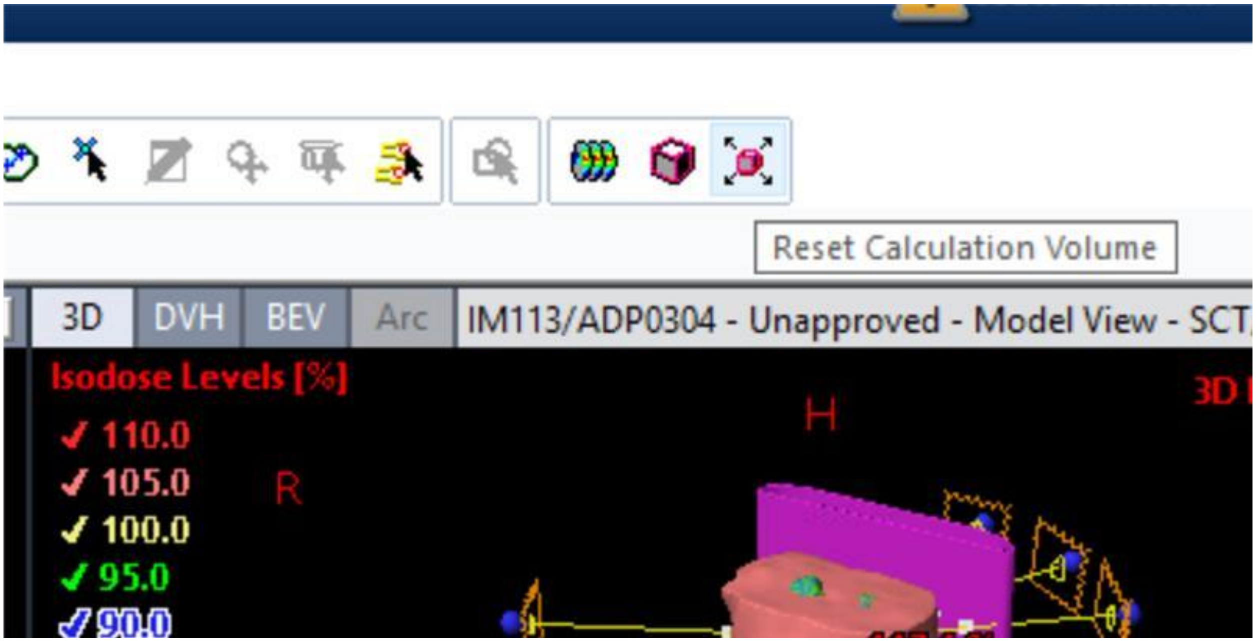

Eclipse Screen Capture – Resetting calculation volume

3. Right click structure set → "Properties" and assign the appropriate Physical Material Table. DO NOT close the Manual Setup Required popup.

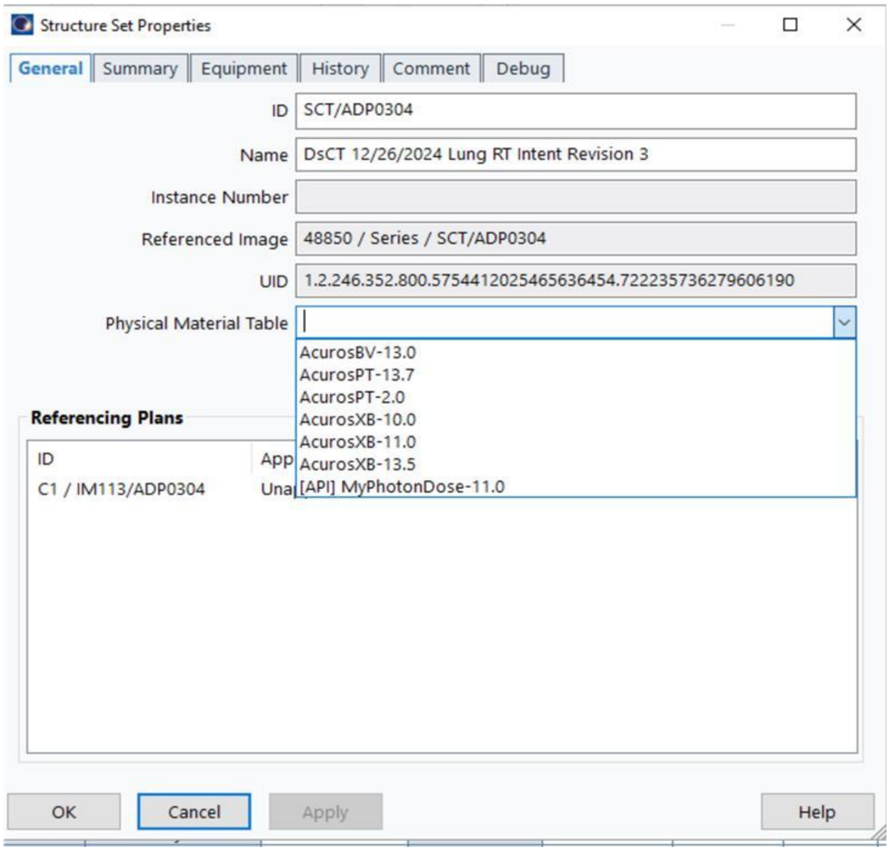

Eclipse Screen Capture – Setting material table

4. Set CT number for “Titanium1”. Click “OK”, close out of properties and click “Save” within eclipse with the GUI still open. Then click “OK”, on the Manual Setup Required popup.

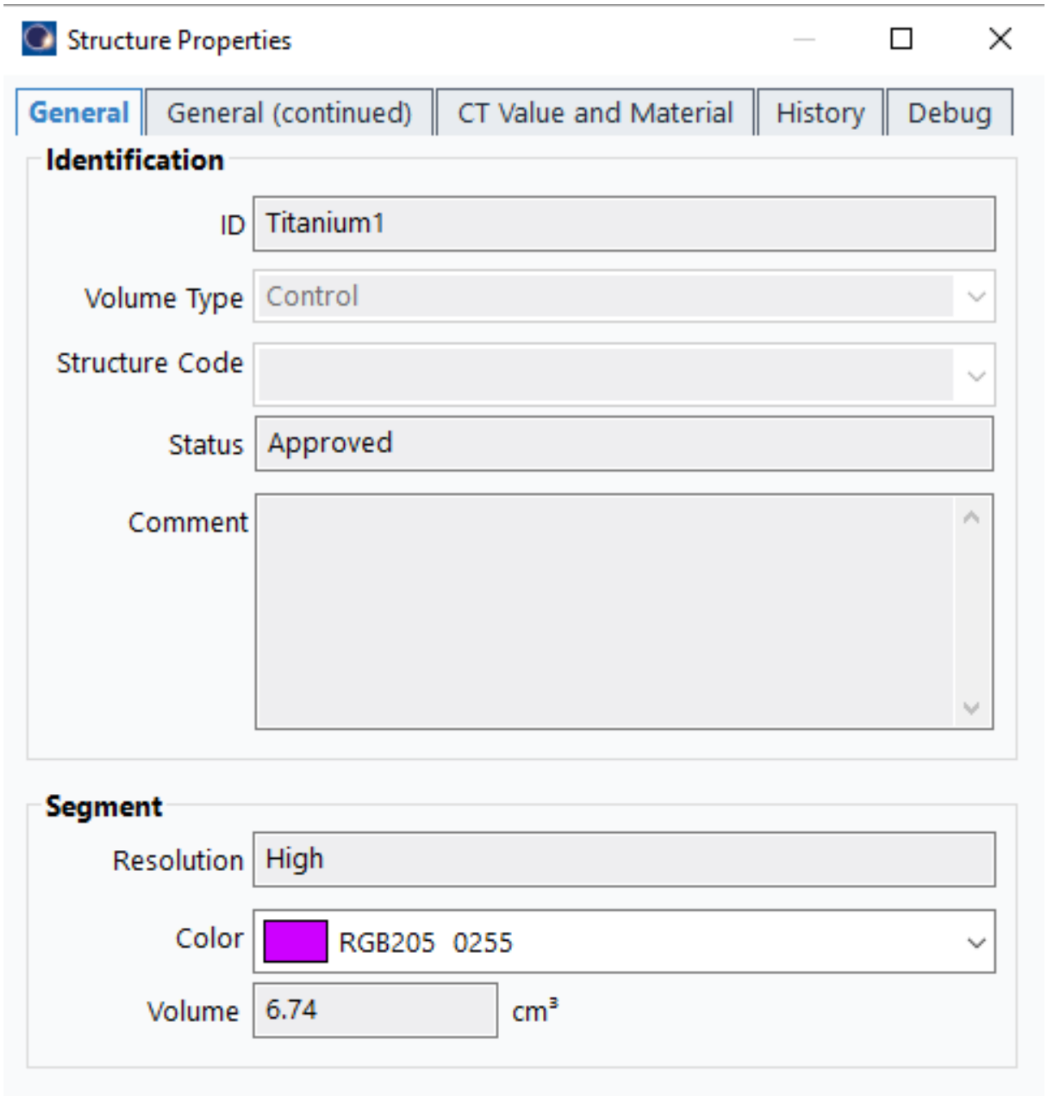

Eclipse Screen Capture – Titanium Override

5. Once you click OK, enter in the remaining number of fractions – Then click “Convert Plan”.

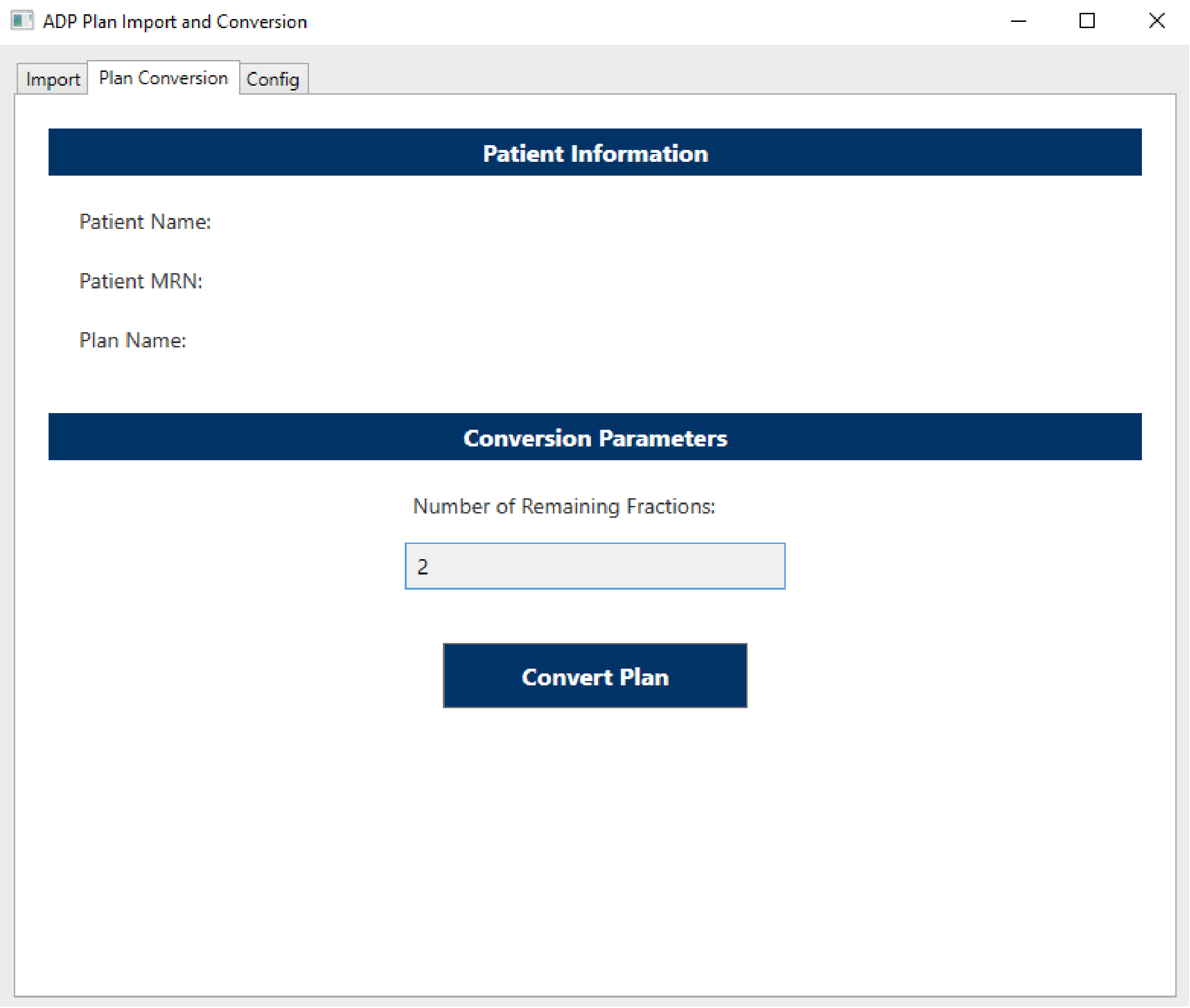

Eclipse Screen Capture – Plan Conversion Tab

6. A pop-up progress window detailing conversion progress will appear – this also serves as a

mechanism for troubleshooting as error messages appear in the detailed windowed below the progress bar.

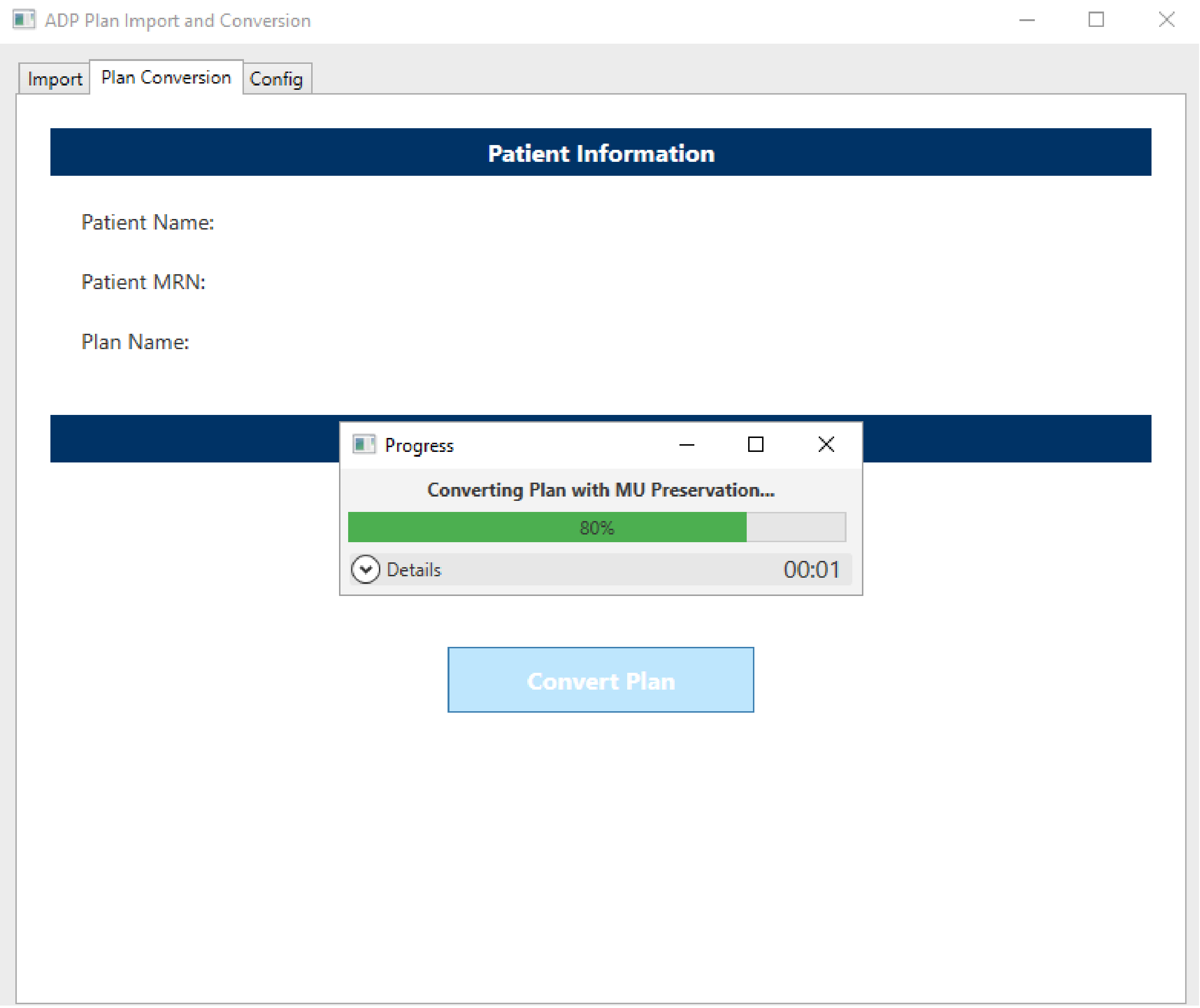

Eclipse Screen Capture –Progress Window

7. A pop-up will appear at the conclusion of the process confirming MU has been maintained

and prompting user to click “OK” and reload patient. You may click OK, close out of GUI and reload patient.

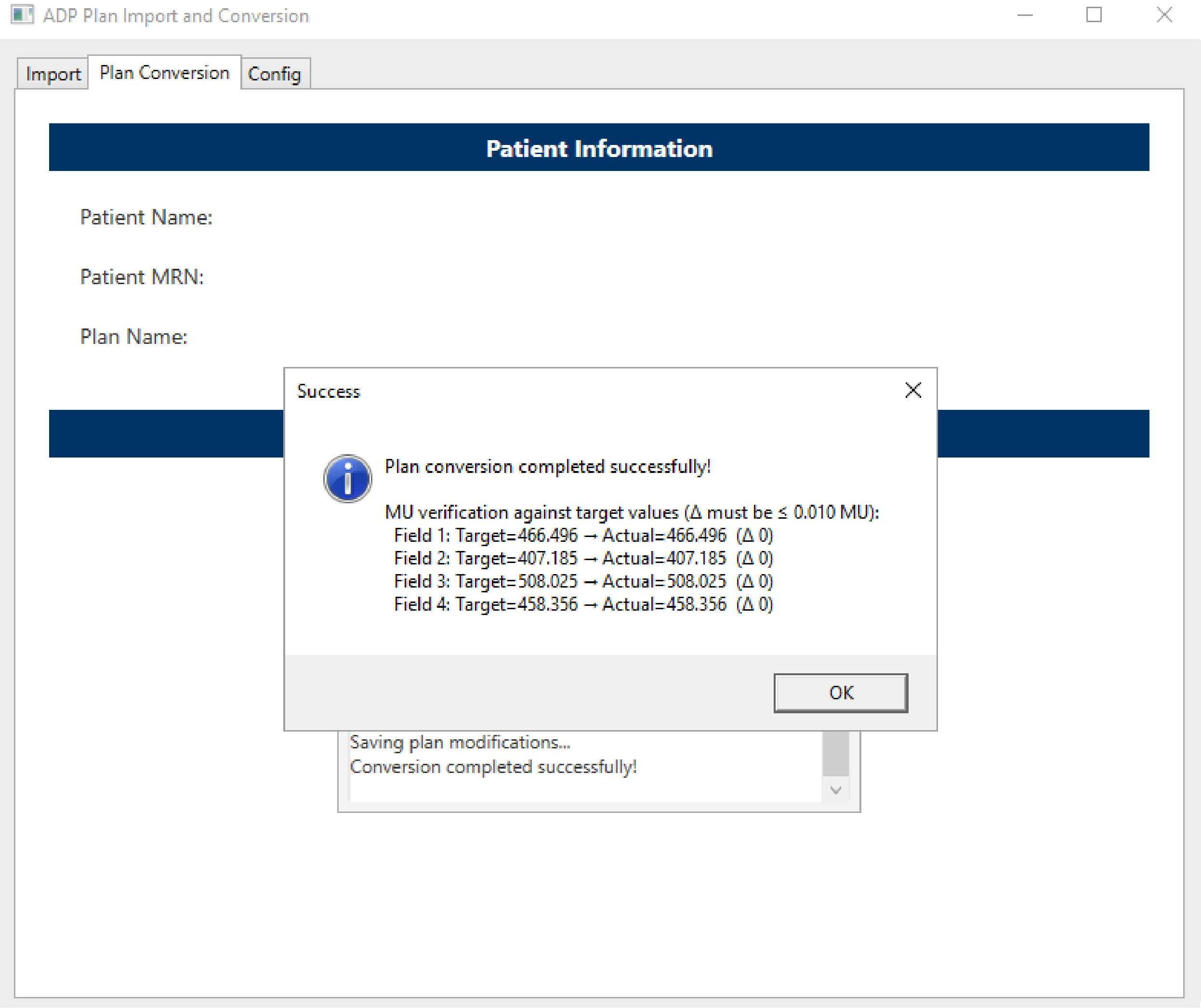

Eclipse Screen Capture – Completion window

Isocenter 1

Position (DICOM): X: 0.00 cm Y: 0.00 cm Z: 0.00 cm
Scale: IEC61217

Field name		Gantry [°] Reference	Gantry [°] Adapted	Collimator [°] Reference	Collimator [°] Adapted	MU Reference	MU Adapted
Field 1	IMRT	179.9°	179.9°	90.0°	90.0°	141.6 MU	132.4 MU
Field 2	IMRT	159.0°	159.0°	80.0°	80.0°	142.5 MU	143.8 MU
Field 3	IMRT	138.0°	138.0°	75.0°	75.0°	112.0 MU	112.5 MU
Field 4	IMRT	116.0°	116.0°	85.0°	85.0°	167.3 MU	130.6 MU
Field 5	IMRT	95.0°	95.0°	355.0°	355.0°	198.7 MU	174.2 MU
Field 6	IMRT	74.0°	74.0°	350.0°	350.0°	142.3 MU	140.2 MU
Field 7	IMRT	53.0°	53.0°	340.0°	340.0°	153.9 MU	144.7 MU
Field 8	IMRT	32.0°	32.0°	5.0°	5.0°	154.6 MU	150.6 MU
Field 9	IMRT	11.0°	11.0°	20.0°	20.0°	198.0 MU	171.5 MU
Field 10	IMRT	349.0°	349.0°	335.0°	335.0°	187.5 MU	162.5 MU
Field 11	IMRT	328.0°	328.0°	330.0°	330.0°	155.3 MU	147.1 MU
Field 12	IMRT	307.0°	307.0°	290.0°	290.0°	141.2 MU	132.5 MU
Field 13	IMRT	286.0°	286.0°	0.0°	0.0°	151.8 MU	142.3 MU
Field 14	IMRT	265.0°	265.0°	7.0°	7.0°	187.5 MU	164.5 MU
Field 15	IMRT	244.0°	244.0°	12.0°	12.0°	194.5 MU	167.3 MU
Field 16	IMRT	222.0°	222.0°	17.0°	17.0°	147.8 MU	153.3 MU
Field 17	IMRT	201.0°	201.0°	25.0°	25.0°	134.4 MU	127.0 MU

Ethos screen capture – Adapted plan MU for Eclipse recalculation verification

8. Set user origin to CT Iso coordinates.
    a. Localize user origin to CT-ISO (Right click CT-ISO structure).
    b. Move viewing planes to structure.
    c. Right click User origin.
    d. Set User Origin.
    e. On dropdown, set to viewing plane intersection and click OK.

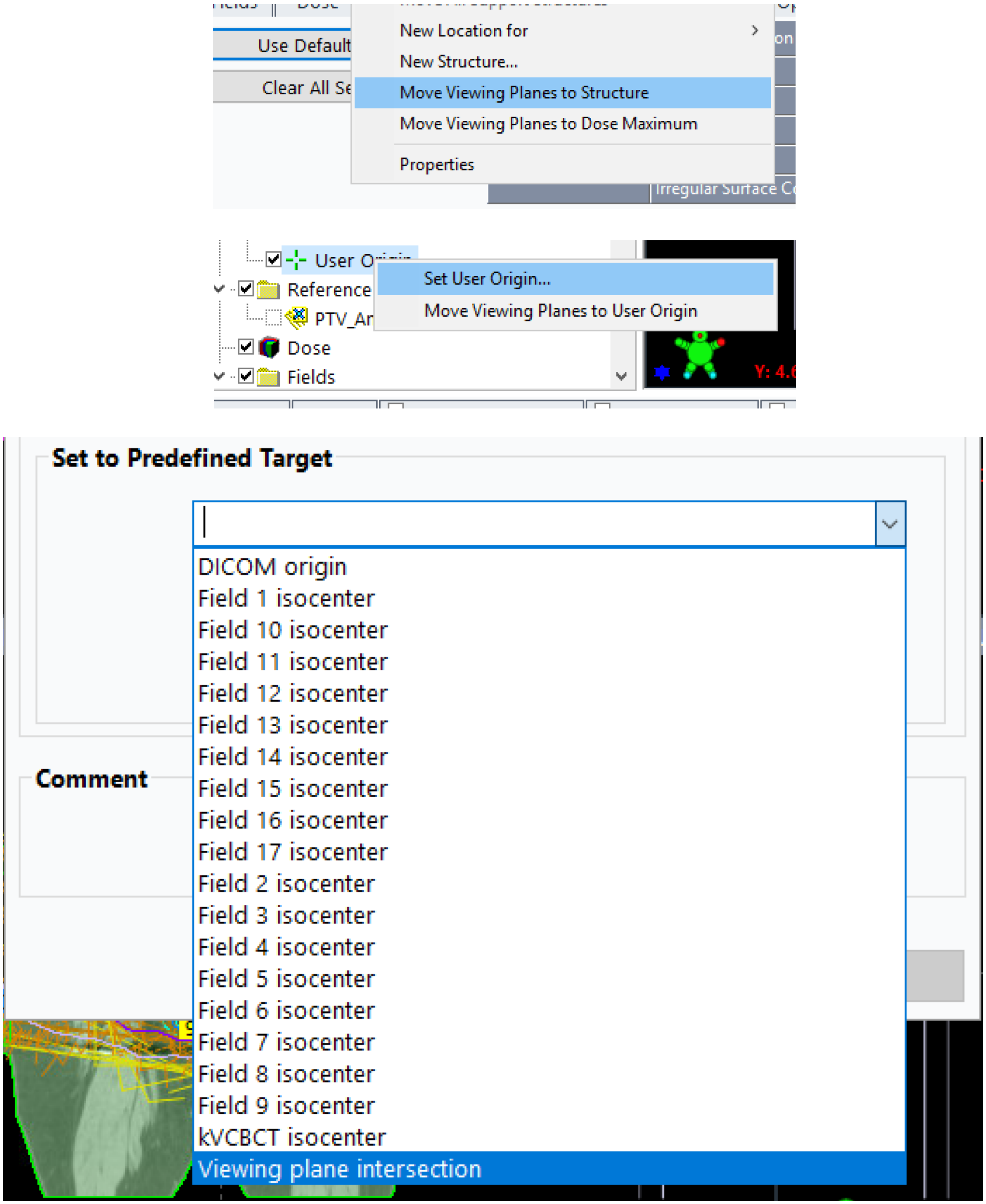

Eclipse screen captures – Setting user origin to CT-Iso coordinates

9. Planning Approved (right click the plan, ‘Plan Approval’, Planning Approved, type in credentials).

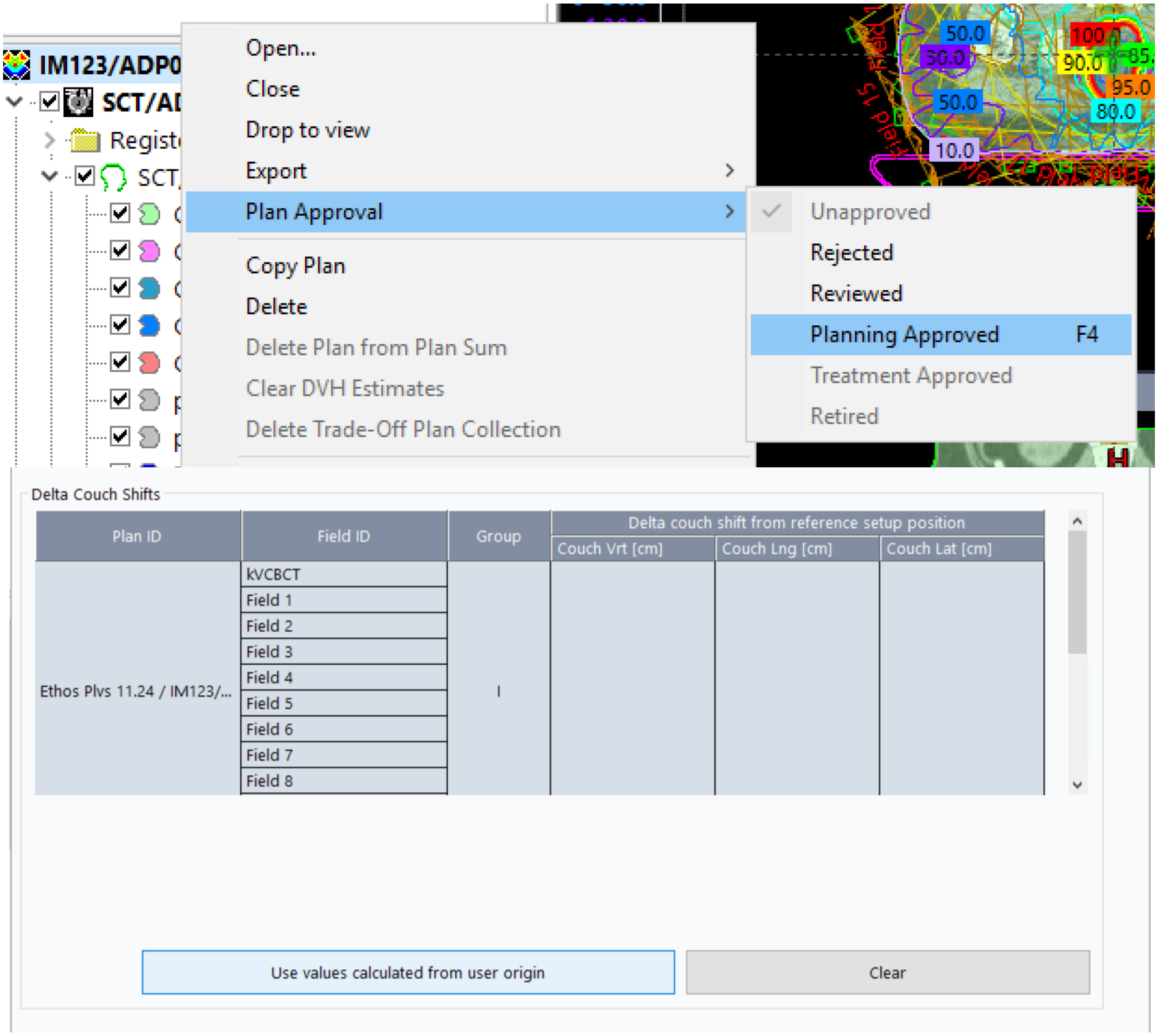

Eclipse screen capture – Plan approval and delta couch shift selection

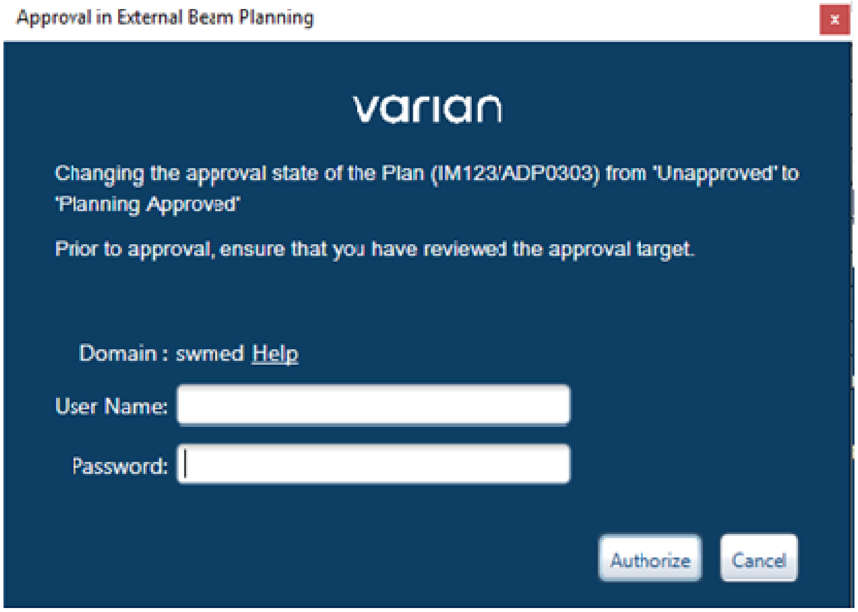

Eclipse screen capture – Approval screen for entering in credentials

CT Isocenter Verification

Purpose: To check that the CT isocenter of the Synthetic CT from the Ethos adapted plan matches original CT isocenter from CT simulation.

Steps:

8. In Eclipse, open the ADP plan in external beam planning and move the viewing plane to the CT-Iso.
9. Navigate to the simulation isocenter location in the technical plan report of the Adapted plan in the Ethos system.
10. Compare the CT-Iso coordinates for reasonable agreement.

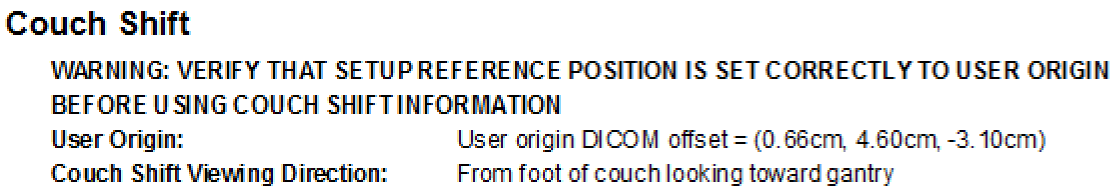

Couch Shift

WARNING: VERIFY THAT SETUP REFERENCE POSITION IS SET CORRECTLY TO USER ORIGIN BEFORE USING COUCH SHIFT INFORMATION

**User Origin:**	User origin DICOM offset = (0.66cm, 4.60cm, -3.10cm)
**Couch Shift Viewing Direction:**	From foot of couch looking toward gantry

Eclipse Plan Report

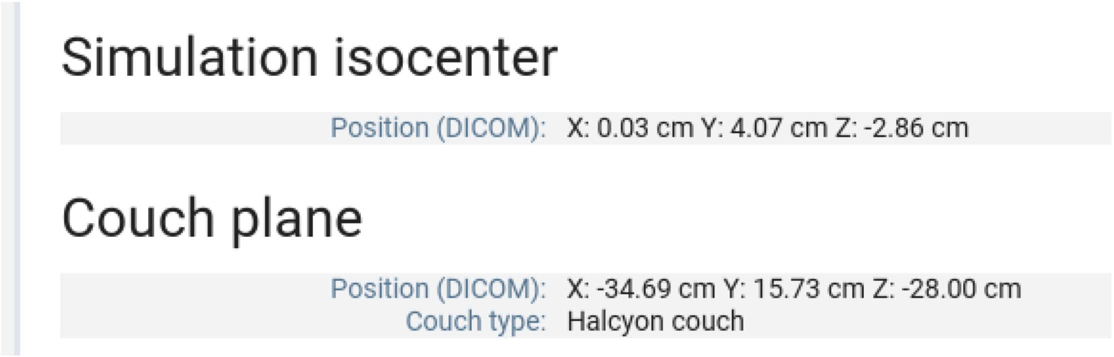

Ethos Technical Plan Report

NOTE: In the event there is no CT isocenter in the ADP plan or the two CT isocenters are not matching:

1. Register the SIM CT to the new synthetic CT for the new plan if no CT isocenter exists.
2. Copy CT isocenter center to the new SCT.

Prescription Association and Plan Scheduling

Purpose: <u>To associate the newly configured plan to the correct prescription and correct the plan schedule in Aria.</u>

Steps:

1. Associate converted plan with correct prescription
   a. Right click approved plan.
   b. Select ‘Properties.
   c. Select ‘RT Prescription’ tab.
   d. Click the drop down arrow and select the correct prescription.

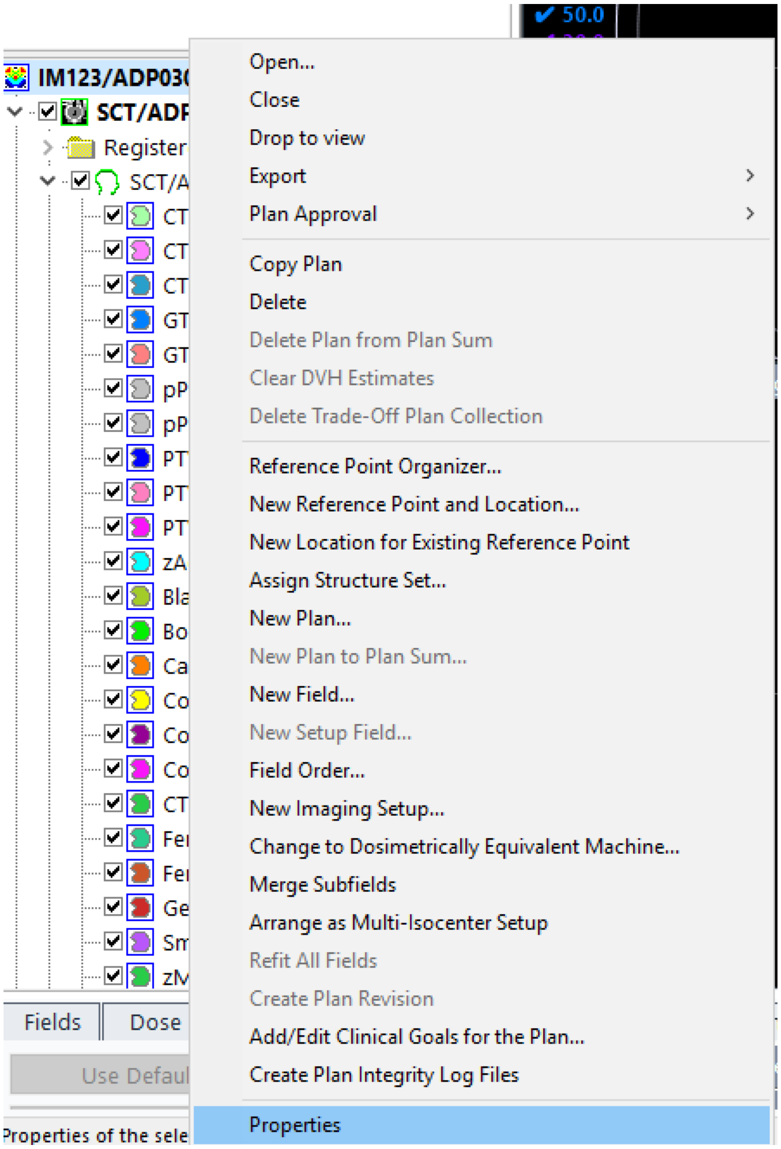

Eclipse screen capture – Selecting ‘Properties’ by right clicking the approved plan

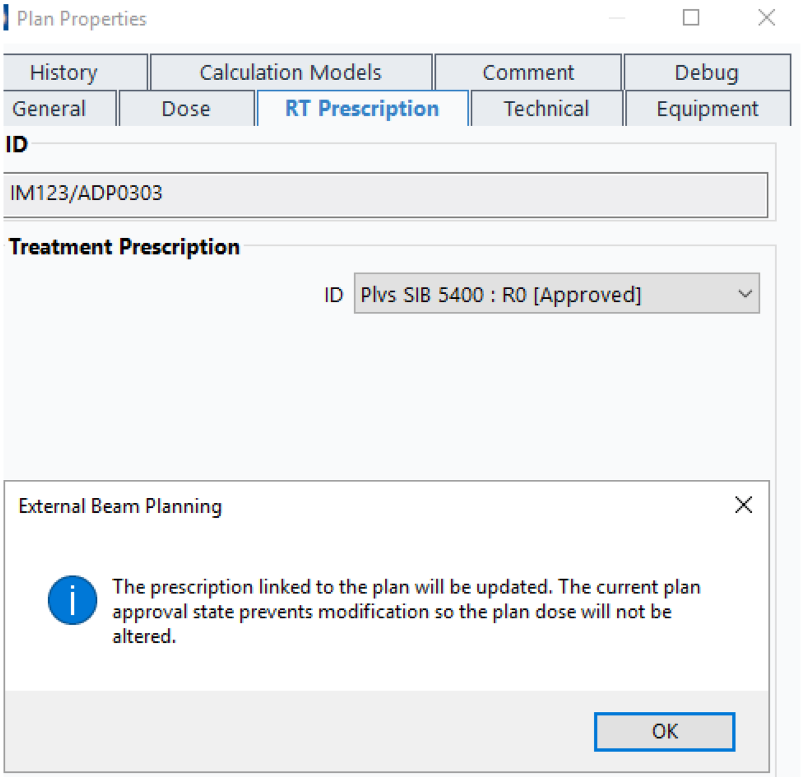

Eclipse screen captures – Associating the correct prescription for the approved plan

2. In the quick links section of Eclipse, Navigate to ‘Plan Scheduling’

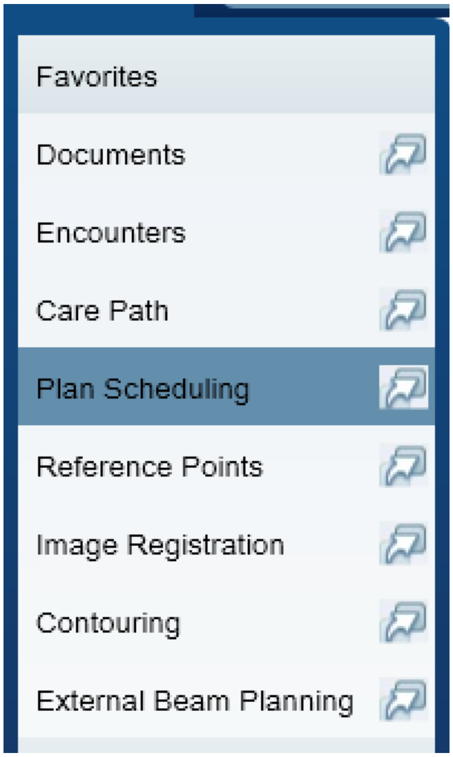

Eclipse screen capture – Navigating to ‘Plan Scheduling’

3. Remove old scheduled fractions and schedule remaining fractions for newly converted plan.
    a. Ensure all of the prior fractions are completed (double check completion of the most recent adaptive fraction).
    b. Remove old plan scheduled fractions.
    c. Schedule remaining fractions for the newly converted plan.

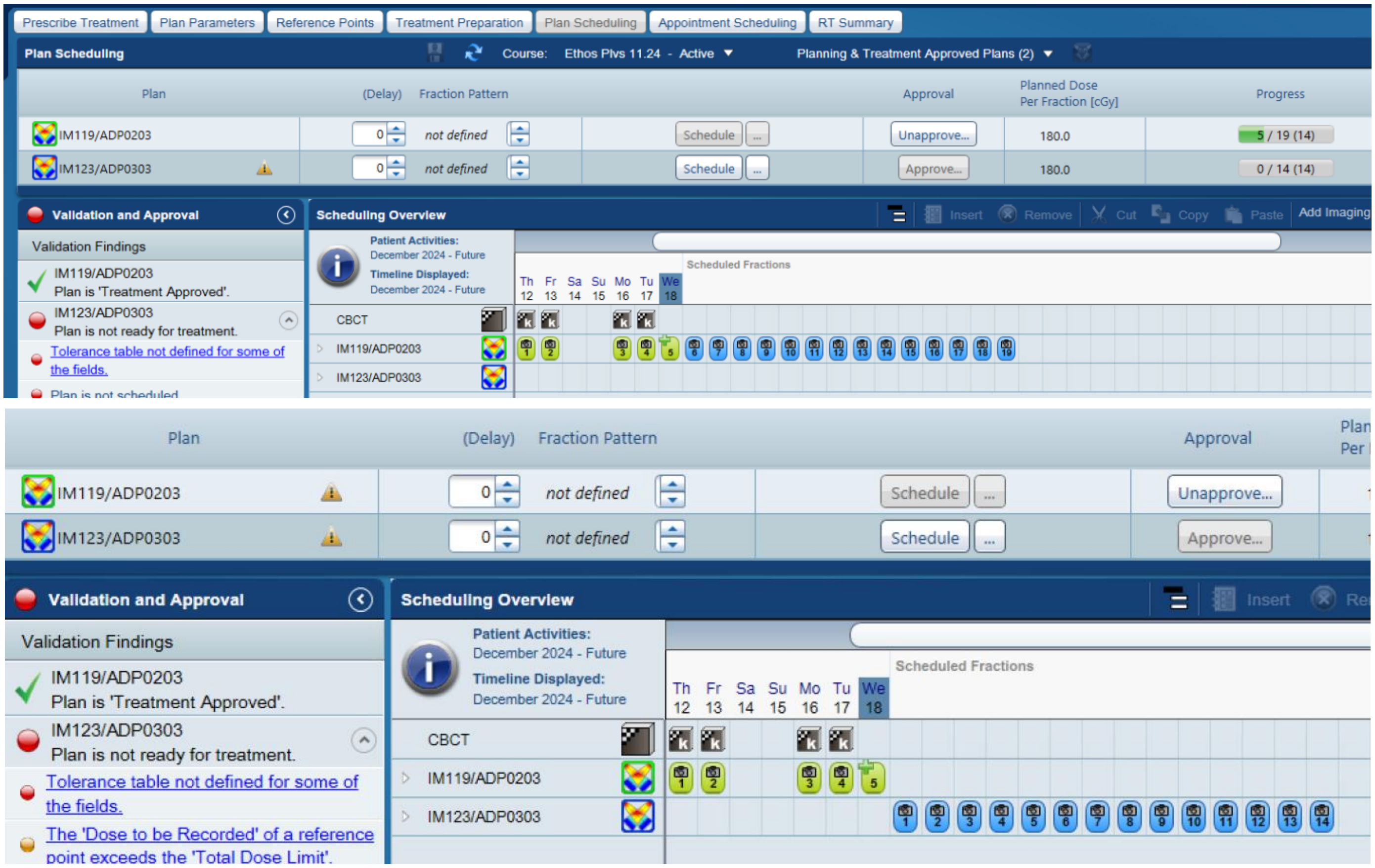

Eclipse screen captures – Top capture → Removal of old plan fractions. Bottom capture → Scheduling of current plan fractions

Plan Preparation and Plan Retirement

Purpose: To set technical parameters of the newly approved and scheduled plan and retirement of old plan.

Steps:

1. In the “Treatment Management’ tab, Navigate to ‘Reference Points’ and setup the dose limits and ensure total dose added up correctly. Below is an example of what this screen looks like.

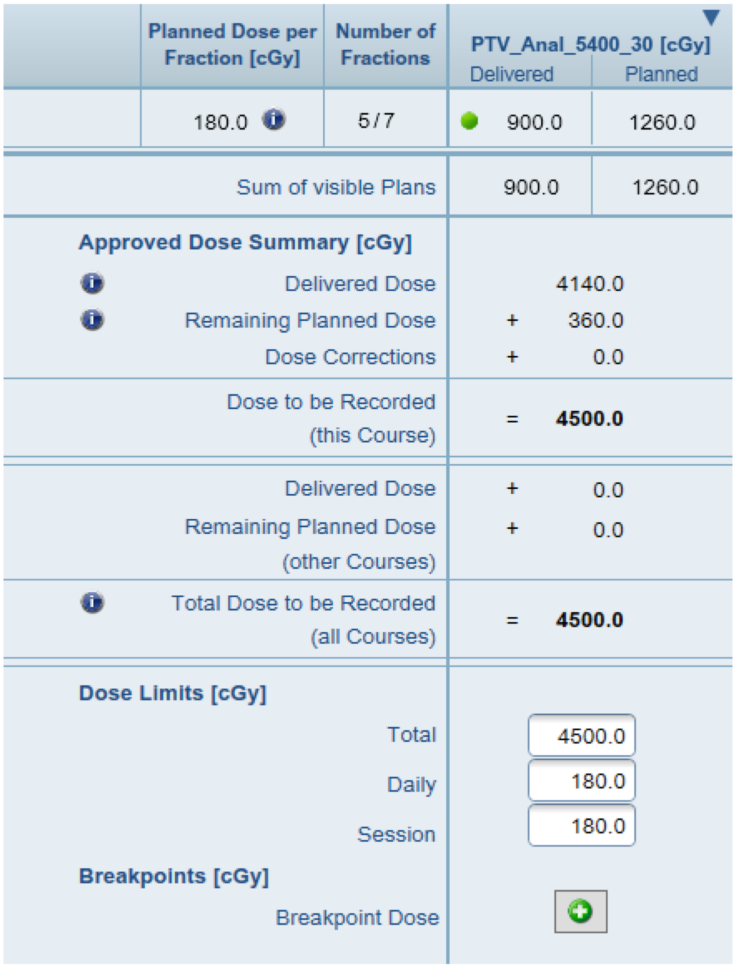

Eclipse screen capture – Reference point dose limits setup

2. In ‘Treatment Preparation’, Setup tolerance table to be whatever is institutionally appropriate. In the below example, this is set to ‘1’.

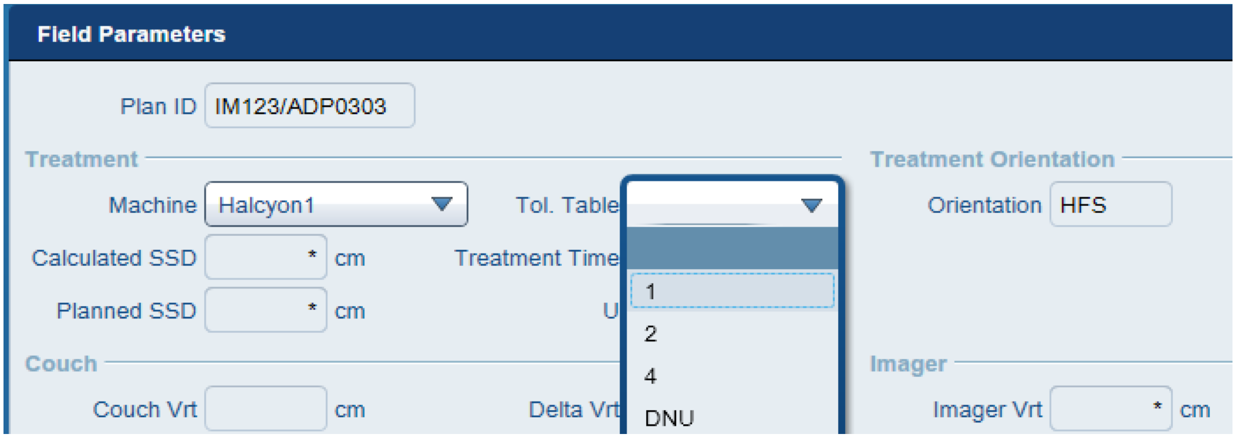

Eclipse screen capture – Setting tolerance table

Note: Although not pictures here, it is also a good idea to make sure the Delta couch shift is intact in this screen.

3. In the “Treatment Preparation” tab establish setup note according to institutional policy. For example, this could be done by copying setup notes from original treatment approved plan to new ADP plan.

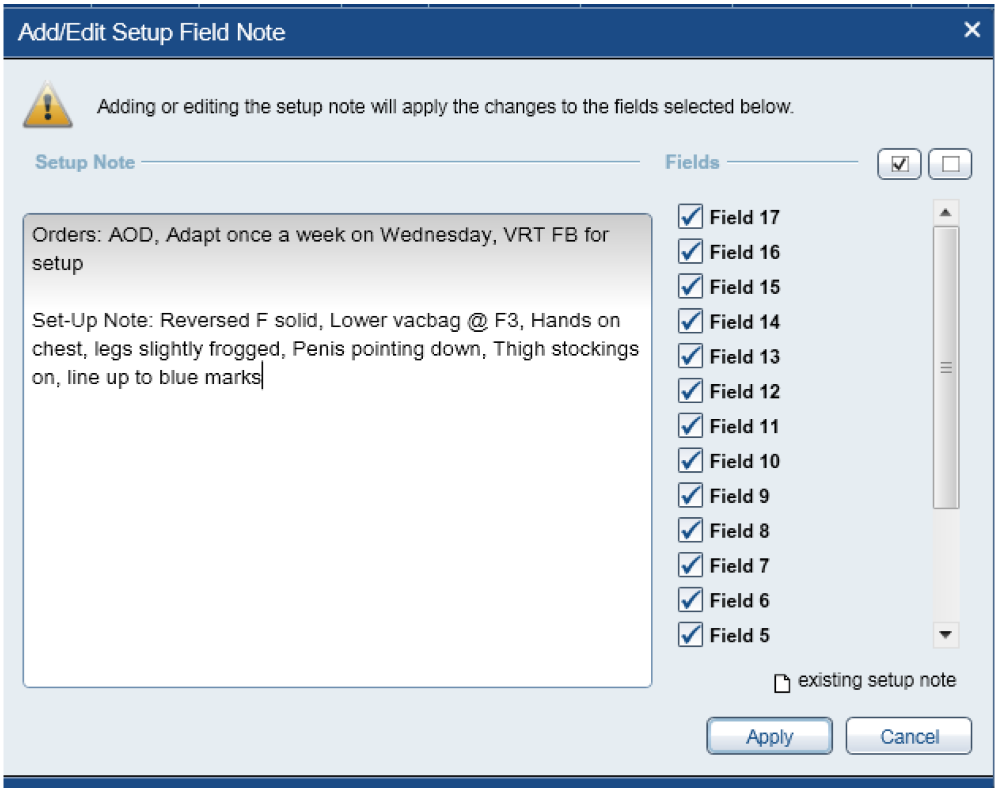

Eclipse screen capture – Adding site setup note to newly converted plan

4. In 'Plan Parameters' → right click on the plan you would like to retire → 'Plan Approval' → 'Completed Early'.

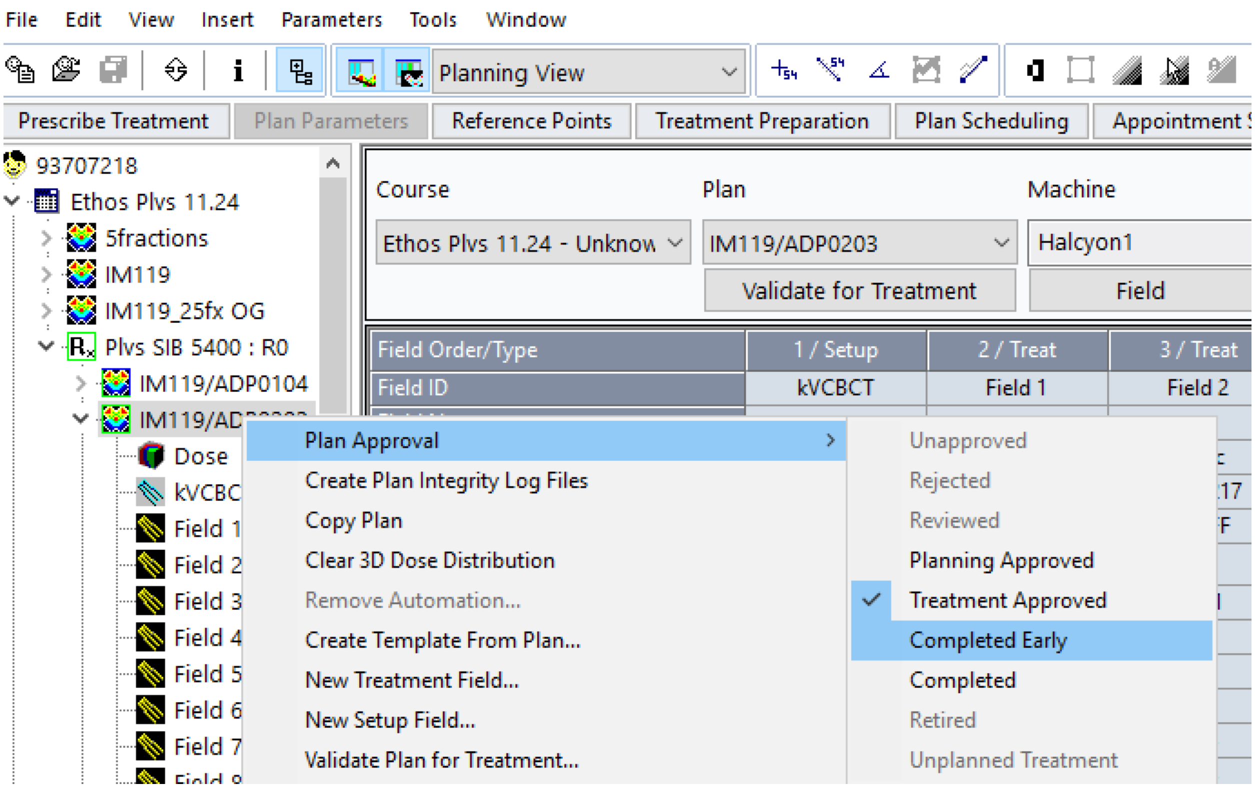

Eclipse screen capture – Retiring old plan by "Completing Early"

Additional Notes

Purpose: To discuss additional components of the plan conversion process outside the scope of this standard operating procedure.

Discussion Items:

1. Plan Printing – As part of this process, institutions may want to print the converted plan and upload the document to Aria for record keeping purposes. The printing process should be set-up and standardized by each respective institution.

2. Plan checking – Institutions may want to consider adding an encounter for this conversion process to help facilitate the conversion process and aid in the 2nd check process. This is an optional step and should be setup and standardized by each respective institution.
